# Mutation of cancer driver *MLL2* results in transcription stress and genome instability

**DOI:** 10.1101/gad.275453.115

**Published:** 2016-02-15

**Authors:** Theodoros Kantidakis, Marco Saponaro, Richard Mitter, Stuart Horswell, Andrea Kranz, Stefan Boeing, Ozan Aygün, Gavin P. Kelly, Nik Matthews, Aengus Stewart, A. Francis Stewart, Jesper Q. Svejstrup

**Affiliations:** 1 Mechanisms of Transcription Laboratory, Clare Hall Laboratories, The Francis Crick Institute, South Mimms EN6 3LD, United Kingdom;; 2 Bioinformatics and Biostatistics Group, The Francis Crick Institute, London WC2A 3LY, United Kingdom;; 3 Biotechnologisches Zentrum, Technische Universität Dresden, 01062 Dresden, Germany;; 4 Advanced Sequencing Facility, The Francis Crick Institute, London WC2A 3LY, United Kingdom

**Keywords:** MLL2, KMT2D, genomic instability, transcription, cancer, mutation

## Abstract

Genome instability is a recurring feature of tumorigenesis. Mutation in *MLL2*, encoding a histone methyltransferase, is a driver in numerous different cancer types, but the mechanism is unclear. Here, we present evidence that *MLL2* mutation results in genome instability. Mouse cells in which *MLL2* gene deletion can be induced display elevated levels of sister chromatid exchange, gross chromosomal aberrations, 53BP1 foci, and micronuclei. Human *MLL2* knockout cells are characterized by genome instability as well. Interestingly, MLL2 interacts with RNA polymerase II (RNAPII) and RECQL5, and, although *MLL2* mutated cells have normal overall H3K4me levels in genes, nucleosomes in the immediate vicinity of RNAPII are hypomethylated. Importantly, *MLL2* mutated cells display signs of substantial transcription stress, and the most affected genes overlap with early replicating fragile sites, show elevated levels of γH2AX, and suffer frequent mutation. The requirement for MLL2 in the maintenance of genome stability in genes helps explain its widespread role in cancer and points to transcription stress as a strong driver in tumorigenesis.

## Abstract (precis)

In this study, Kantidakis et al. investigate the mechanism underlying the role of MLL2 in tumorigenesis and find that MLL2 mutation causes genome instability. They show that MLL2 mutated cells display signs of substantial transcription stress, and the most affected genes overlap with early replicating fragile sites, show elevated levels of γ-H2AX, and suffer frequent mutation.

It has become increasingly clear that transcription, an essential and tightly regulated process, is associated with genome instability (Aguilera and Gomez-Gonzalez 2008; Svejstrup 2010). The mechanisms are still poorly understood, but transcription-associated genome instability is at least partly caused by clashes with the replication fork, resulting in mutagenesis or chromosomal breaks that can lead to genome reorganization (Bermejo et al. 2012; Kim and Jinks-Robertson 2012; Helmrich et al. 2013). Since the acquisition of genomic instability is a pivotal event during tumorigenesis (Hanahan and Weinberg 2011), understanding the mechanisms that maintain genome integrity during transcription is of great importance.

We recently reported that RECQL5, a member of the RECQ family of helicases, is a general RNA polymerase II (RNAPII) elongation factor that is important for preserving genomic stability during transcription (Saponaro et al. 2014). Indeed, depletion of RECQL5 results in increased pausing, stalling, arrest, and/or backtracking (collectively referred to as transcription stress) at sites that overlap with regions of genome instability. RECQL5 serves as an interesting example of proteins that function at the interface between several cellular processes, such as transcription, DNA replication, and recombination, to maintain genome integrity.

Chromatin poses a substantial challenge for the transcribing RNAPII. As it transcribes through nucleosomes, RNAPII interacts with chromatin-modifying enzymes, affecting chromatin structure and nucleosome displacement/reassembly. In the absence of tightly regulated cotranscriptional chromatin rearrangement, transcript elongation can be interrupted, resulting in transcription stress and potential disturbance of the integrity of the transcribed region (Svejstrup 2007; Selth et al. 2010; Saponaro et al. 2014). In light of these considerations, it is intriguing that mutations in chromatin-regulating enzymes have been identified in several recent cancer genome-sequencing projects (for recent reviews, see Roy et al. 2014; Van Rechem and Whetstine 2014). For example, the gene encoding *MLL2*, a histone H3 Lys4 (H3K4) methyltransferase (HMT), is mutated in a large number of different cancers. These include diffuse large B-cell lymphoma (Pasqualucci et al. 2011), follicular lymphoma (Okosun et al. 2014), medulloblastoma (Parsons et al. 2011; Pugh et al. 2012), pediatric cancers (Huether et al. 2014), breast cancer (Stephens et al. 2012), lung carcinomas (The Cancer Genome Atlas Research Network 2012; Ross et al. 2014; Yin et al. 2014), parathyroid carcinoma (Kasaian et al. 2013), esophageal squamous cell carcinoma (Gao et al. 2014; Song et al. 2014), head and neck squamous carcinomas (Seiwert et al. 2015), renal carcinoma (Dalgliesh et al. 2010), urothelial bladder carcinoma (Balbas-Martinez et al. 2013), and prostate cancer (Grasso et al. 2012).

MLL2 (also known as ALR or KMT2D; human gene ID 8085; originally called MLL4 in mice) is the largest member (594 kDa) of the Trithorax-type HMTs (Ruthenburg et al. 2007). In mammals, this group of enzymes also includes SETD1A, which appears to be responsible for the majority of cellular H3K4 trimethylation (H3K4me3) (Bledau et al. 2014), as well as MLL1, MLL3, and MLL4. MLL1 has been extensively studied due to its well-established role in leukemia (Li and Ernst 2014), but less is known about the other members of the group. The closely related proteins MLL2 and MLL3 reside in nearly identical complexes, termed ASCOM (Lee et al. 2009). Recent studies have provided evidence for a role for MLL2 and MLL3 in histone H3K4 methylation at enhancers (Hu et al. 2013; Lee et al. 2013; Cheng et al. 2014), and it has thus been suggested that their role in cancer might be explained through deregulated expression of oncogenes and tumor suppressors (Herz et al. 2014). However, the transcriptome of cells lacking MLL2 shows remarkably few differences from that of the wild-type counterpart, leaving few clues to explain tumorigenesis this way (Issaeva et al. 2007; Guo et al. 2012).

In the process of generating interactomes for a number of transcription proteins, we observed that MLL2 associates with RECQL5, prompting us to investigate whether MLL2 might play a role in maintaining genome stability in genes. Here we report that *MLL2* mutation indeed gives rise to significant genomic instability in areas overlapping with early replicating fragile sites (ERFSs). This may be explained by the involvement of MLL2 in transcript elongation and specifically in mediating elongation-associated histone H3K4 methylation. Indeed, cells mutated for *MLL2* display significant transcription stress in short, highly active genes, which overlap with the sites of genome instability. Together, these findings can potentially explain the surprisingly widespread involvement of MLL2 in human cancer.

## Results

### MLL2 interacts with RECQL5

A human cell line expressing a Flag-tagged version of the RECQL5 protein (Aygun et al. 2008) was used to perform affinity purification experiments followed by mass spectroscopy. This established that RECQL5 associates with RNAPII subunits and several transcription-related factors, including elongation factors (SPT5 and SPT6), and the Mediator and Integrator complexes (Supplemental Fig. S1; Supplemental Table S1). These results complemented previous data showing that RECQL5 interacts with the elongating form of RNAPII (Aygun et al. 2008). Unexpectedly, however, we also detected MLL2 with high confidence (Supplemental Fig. S1A). Subsequent high-sensitivity mass spectroscopy analysis confirmed the interaction between RECQL5 and MLL2, and several other components of the MLL2 complex were now also identified, including the PTIP subunit (see Fig. 5, below; Supplemental Fig. S1B; Supplemental Table S2). Notably, we consistently failed to detect MLL3 in these experiments.

### Mouse cells defective for MLL2 present genomic instability

RECQL5 has a well-established role in maintaining genomic stability (Aygun and Svejstrup 2010; Saponaro et al. 2014). While the mechanistic relevance of MLL2–RECQL5 interaction is yet to be investigated, the connection spurred us to investigate the potential role of MLL2 in the maintenance of genome integrity. For this purpose, we initially used immortalized mouse embryonic fibroblasts (MEFs) in which both *MLL2* alleles were targeted by loxP (F/F cells). Addition of tamoxifen to these cells for 24 h resulted in the complete excision of *MLL2* exons 3–5 (hereafter referred to as FC/FC cells), loss of mRNA over the targeted regions, and a change in reading frame that inserts a stop codon, as designed (Supplemental Fig. S2).

**Figure 1.**
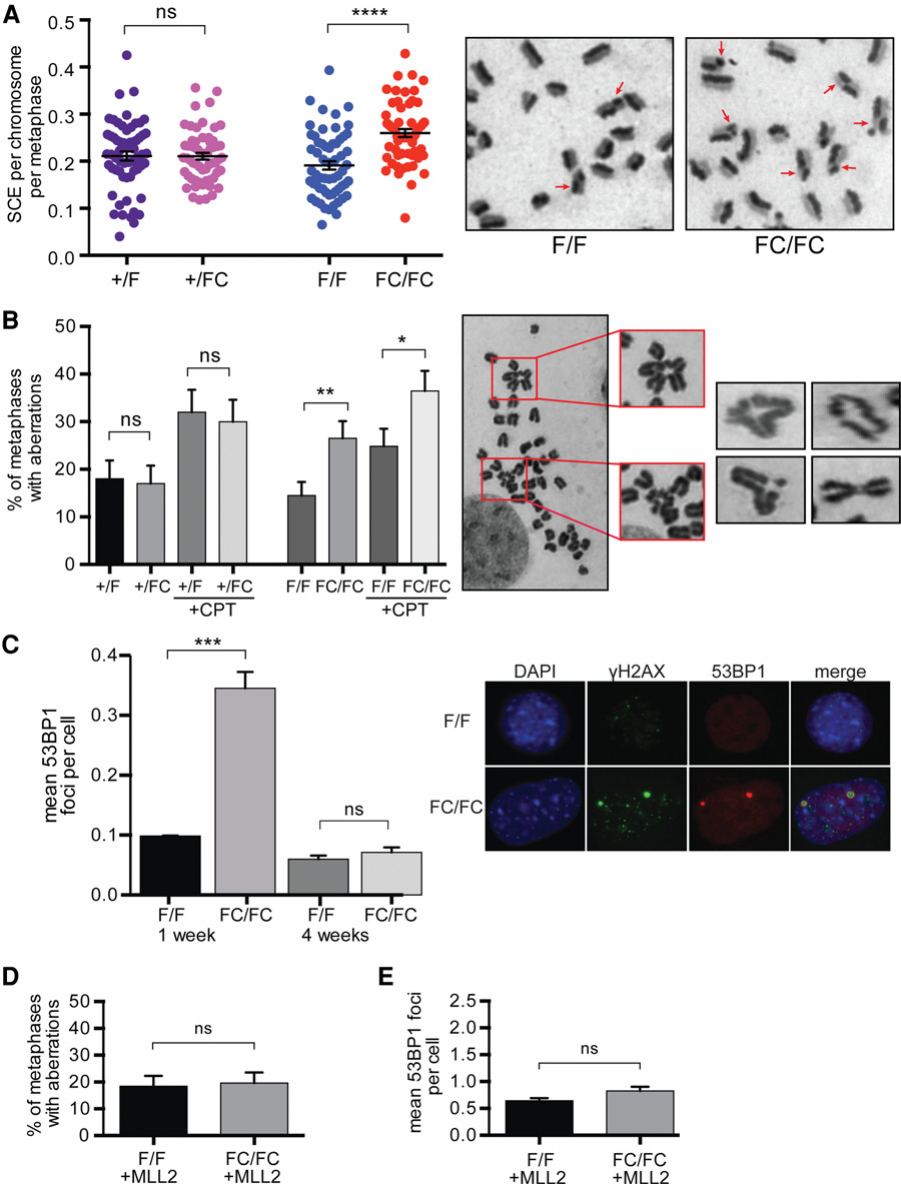
Genomic instability phenotypes in *MLL2* FC/FC mouse MEF cells. (*A*) Sister chromatid exchange quantification. Each dot represents a metaphase. *n* = 60 metaphases for each cell line. The red arrows indicate sister chromatid exchange events. (*B*) Gross chromosomal aberration (GCA) quantification in the presence or absence of camptothecin (CPT). Representative examples of aberrations are shown in the images at the *right*. *n* ≥ 100 metaphases for each cell line/treatment. (*C*) Immunofluorescence using antibodies against 53BP1 (red) and γH2AX (green). Nuclei are shown in blue. *n* = 3 independent experiments scoring >500 cells in total for each cell line. (*D*) GCA quantification in MLL2 BAC rescue experiments. *n* ≥ 100 metaphases from two different *MLL2* bacterial artificial chromosome (BAC) clones for each cell line. (*E*) 53BP1 quantification in *MLL2* BAC rescue experiments. *n* = 6 independent experiments from two different *MLL2* BAC clones scoring >1500 cells in total for each cell line. *P*-values were determined by Mann-Whitney test. Error bars represent SEM. For all figures in this study, *P* < 0.05 (*), *P* < 0.01 (**), *P* < 0.001 (***), *P* < 0.0001 (****), and not significant (ns).

*MLL2* FC/FC and control cells were used in a variety of assays to test the hypothesis that MLL2 affects genome integrity. First, we measured sister chromatid exchange in *MLL2* FC/FC and the parental F/F cells as well as independently derived +/FC control cells and their parental +/F cells. The latter cells were included to exclude the possibility that expression of Cre recombinase in itself had an effect. Interestingly, although sister chromatid exchange was measured after only a limited number of cell doublings after *MLL2* excision, the FC/FC cells presented significantly higher levels of exchange than parental cells, while this was not the case for the +/FC control cells (Fig. 1A).

We next looked at gross chromosomal aberrations (GCAs), including radial chromosomes, chromosomal fusions, and chromatid breakages. The FC/FC cells presented significantly higher levels of GCAs than the paternal cells, at a level similar to that of cells treated with camptothecin (CPT), a topoisomerase 1 inhibitor that generates DNA damage. Not surprisingly, the number of GCAs in the FC/FC cells increased even further after treatment with CPT (Fig. 1B).

If manipulation of MLL2 indeed results in DNA lesions, then these might be marked by phosphorylation of H2AX (a form designated γH2AX) (Rogakou et al. 1998), while 53BP1 nuclear bodies (Lukas et al. 2011) might also be manifested. Indeed, elevated levels of 53BP1 foci that colocalized with γH2AX were frequently detected in FC/FC cells (Fig. 1C, right panel). Using automatic recognition software for quantification (Carpenter et al. 2006), a threefold or greater increase in 53BP1 foci was observed in the FC/FC cells 1 wk after *MLL2* excision (Fig. 1C, left panel). No difference was detected between +/FC and +/F cells (Supplemental Fig. S3A). Interestingly, the increase in 53BP1 foci in FC/FC cells was only transient, as the levels were consistently indistinguishable from the controls when cells were instead grown for 4 wk (Fig. 1C, left). We also scored the cells for the presence of micronuclei as another sensitive indicator of genome instability. A marked increase of micronuclei was observed in FC/FC cells, which, like the increase in 53BP1 foci, was transient (Supplemental Fig. S3B). As expected, control cells showed no significant change (Supplemental Fig. S3C). A trivial explanation for the transient nature of some of these changes might be if a subgroup of cells failed to excise *MLL2* and were then clonally expanded during subsequent cell culture. However, *MLL2* loss was similarly complete in cells grown for 1 wk and 1 mo (data not shown). This suggests that cells become genomically unstable immediately after the loss of MLL2 function but somewhat stabilize with time so that only some features of genome instability remain observable.

Similar to our previous analysis of RECQL5 (Saponaro et al. 2014), we also used comparative genomic hybridization (CGH) to detect genomic regions that were gained or lost as a result of disabling MLL2 function. A limited number of recurring events was identified (Supplemental Fig. S4A), which occurred independently in two experiments performed 1 or 4 wk after *MLL2* excision. We confirmed by γH2AX chromatin immunoprecipitation (ChIP) that the regions encompassing the break sites detected by CGH were indeed subject to genomic alteration/damage (Supplemental Fig. S4B).

Together, the data above provide compelling evidence that *MLL2* mutation results in genome instability. To confirm that the cellular defects above were due to loss of MLL2 function, we used a stable F/F cell line carrying a bacterial artificial chromosome (BAC) expressing wild-type MLL2, as we could not consistently and convincingly detect the ∼600-kDa MLL2 protein by Western blotting. The resulting cell line was treated with tamoxifen to excise the floxed MLL2 alleles but leave the BAC-encoded allele intact. Gratifyingly, FC/FC cells expressing MLL2 from the integrated BAC had levels of GCAs and 53BP1 foci that were indistinguishable from the parental cells (Fig. 1, cf. D,E and B,C, respectively).

We conclude that mutation of *MLL2* in mouse cells results in genomic instability.

### Human cells defective for MLL2 show genome instability as well

**Figure 2.**
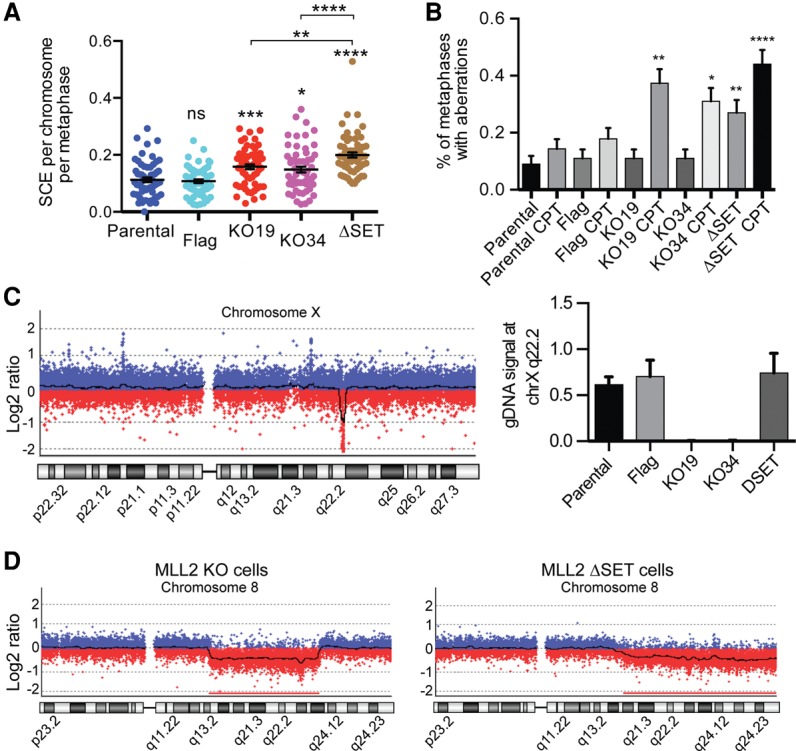
MLL2 maintains genomic stability in human HCT116 cells. (*A*) Sister chromatid exchange quantification. The parental and *MLL2* Flag-tagged cells were used as controls. Each dot represents a metaphase. *n* = 60 metaphases for each cell line. (*B*) GCA quantification in the presence or absence of CPT. *n* ≥ 100 metaphases for each cell line or treatment. (*C*, *left*) CGH profile of chromosome X (chrX) in MLL2 KO19 cells compared with the parental line. The *COL4A5/6* locus at q22.3 is lost. (*Right*) Genomic DNA PCR for the *COL4A5/6* locus. (*D*) CGH profile of chromosome 8 (chr8) in MLL2 KO19 and ΔSET cells compared with the parental line. Error bars represent SEM. *P*-values were determined by Mann-Whitney test.

We also investigated whether MLL2 affects genome stability in human cells. For this purpose, we used two independently derived *MLL2* knockout cell lines: KO19 and KO34. As controls, the parental HCT116 line as well as a *MLL2*-*Flag* wild-type cell line were used (all were from Guo et al. 2012). As expected from the experiments with mouse cells, human cells lacking MLL2 had higher levels of sister chromatid exchange compared with the two control cell lines (Fig. 2A). The human *MLL2* knockout cells did not show GCAs under normal conditions but were significantly more sensitive than the controls to treatment with CPT (Fig. 2B). We also measured 53BP1 foci in the human *MLL2* knockouts but found no significant difference (Supplemental Fig. S3D). Given that these human cells are stable knockout lines, this was not unexpected, as the number of 53BP1 foci were also only transiently elevated in the mouse *MLL2* FC/FC cells (see Fig. 1C). In general, these experiments are consistent with the idea that *MLL2* mutation promotes transient and substantial genome instability, which, to some extent, stabilizes with time.

We also hypothesized that since the human *MLL2* knockouts are stable cell lines that have undergone several divisions without MLL2, copy number changes in genomic loci might have accumulated to a higher level than that observed in the mouse cell lines. We therefore again performed CGH experiments, this time comparing the two independently derived human *MLL2* knockouts with the parental or Flag-tagged cell lines. We detected 18 aberrations (11 gains and seven losses) that were detected in either one or both knockout lines, compared with the controls (Supplemental Fig. S5A).

Gene expression microarray analyses using the same cell lines have already been published, and only relatively few genes are affected by *MLL2* deletion (Guo et al. 2012). Given that several different genomic regions are lost or gained with a high frequency in the human *MLL2* knockout cells, we compared the list of genes whose expression was affected by *MLL2* deletion (Guo et al. 2012) with the genes that were affected by the genomic gains or losses in our CGH assays. Remarkably, close to one out of five of the genes with lower expression in *MLL2* knockout cells are located in genomic regions that were lost, while approximately one in 10 of the genes with higher expression are in genomic regions that were called as gains in our CGH analysis. The gene with the lowest level of expression in *MLL2* knockout cells, *COL4A5* (Guo et al. 2012), was actually completely lost in both *MLL2* knockout cells lines, as confirmed by quantitative PCR (qPCR) of genomic DNA (Fig. 2C). These experiments show that lack of MLL2 can result in losses or gains of genomic loci, which in turn may affect the transcriptome and be misinterpreted as reduced levels of transcription by gene expression analysis.

We next investigated whether the SET domain, the catalytic methyltransferase domain of MLL2, might be important for maintaining genome stability. To this end, we again used a human cell line derived by the He laboratory (Guo et al. 2013) that expresses a form of MLL2 lacking the SET domain, located at the extreme C-terminal of the protein (ΔSET). Remarkably, sister chromatid exchange assays showed not only that ΔSET cells had a significantly higher number of exchange events than the parental and Flag-tagged control cells but that genomic instability was significantly increased even compared with the *MLL2* knockout cell lines (Fig. 2A). Moreover, we found that, in contrast to the knockout cells, the metaphase chromosomes of the ΔSET cells had a significantly elevated number of abnormalities even in the absence of CPT, which was further increased by CPT treatment (Fig. 2B). Together, these data indicate that cells expressing MLL2 lacking the SET domain show even more genome instability than cells expressing no MLL2 at all.

To further expand on this finding, we performed CGH experiments to map gains and losses in the ΔSET cells. Twenty-nine aberrations (15 losses and 14 gains) were uncovered (Supplemental Fig. S5B). Interestingly, half of the aberrations (nine of 18) found in the knockout cell lines were also detected in the ΔSET cells. On the other hand, certain aberrations, such as the loss of *COL4A5* (Fig. 2C), were not observed in the ΔSET cells, and vice versa. Moreover, some regions, such as on chromosome 8, were lost to an even larger extent in ΔSET cells than in the knockout cell lines (Fig. 2D).

Together, the data presented thus far indicate that MLL2 is important for maintaining genome stability in both mouse and human cells and that loss of its SET domain strongly affects the process.

### Elevated levels of transcription-associated DNA damage in MLL2 mutants

The data above establish that *MLL2* mutation results in pronounced genome instability, but the underlying mechanism remained unclear. We first considered the possibility that genome instability in the *MLL2* mutated cells might be brought about indirectly; for example, through deregulation of repair genes. However, *MLL2* mutation had a barely discernable effect on gene expression in our mouse cells, as previously observed in human cells (Issaeva et al. 2007; Guo et al. 2012). Indeed, we found that only 37 genes showed increased expression, while 55 had decreased expression (greater than twofold) in *MLL2* FC/FC mouse cells (Supplemental Table S3). Importantly, none of the encoded proteins are involved in the maintenance of genome stability.

**Figure 3.**
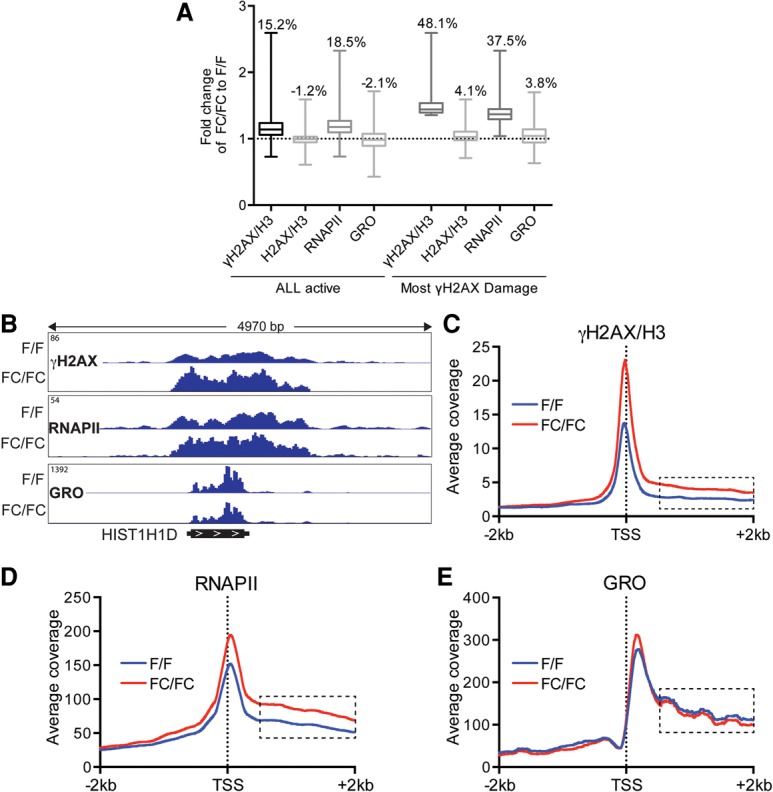
RNAPII ChIP-seq, γH2AX ChIP-seq, and genome-wide nuclear run-on sequencing (GRO-seq) profiles in MLL2 mutated cells. (*A*) Fold change variation in γH2AX/H3, H2AX/H3, RNAPII ChIP-seq density levels and GRO-seq levels over the gene body ±500 base pairs (bp) in FC/FC versus F/F cells in all actively transcribed genes or genes with the most DNA damage, as indicated. The whiskers denote minimum to maximum. The depicted arithmetic values represent geometric mean differences. (*B*) ChIP-seq profiles over the *HIST1H1D* gene for γH2AX, RNAPII, and GRO-seq in F/F and FC/FC cells. Profiles of γH2AX (normalized to H3) (*C*), RNAPII (*D*), or GRO (*E*) for the genes with the most DNA damage.

To try to identify specific sites of genome instability in *MLL2* FC/FC cells, we performed genome-wide analysis with γH2AX as a proxy for DNA damage and H2AX and H3 as controls. As previously noted by others (Iacovoni et al. 2010; Seo et al. 2012), γH2AX was enriched in transcribed regions and depleted from intergenic regions (Supplemental Fig. S6A,B). We therefore focused our further analysis on genes. Importantly, many genes had significantly higher levels of γH2AX in *MLL2* FC/FC cells. As a matter of fact, the most affected genes (414; 10% of total) had almost 50% higher γH2AX density, on average, in *MLL2* FC/FC cells than in wild type (Fig. 3A; Supplemental Table S4). The validity of the ChIP sequencing (ChIP-seq) data were confirmed by ChIP-qPCR experiments for several genes (Supplemental Fig. S6C), and we also confirmed the γH2AX occupancy data using a distinct anti-γH2AX antibody (Supplemental Fig. S6D). More modest average increases for γH2AX occupancy were observed when all active genes were computed (Fig. 3A, all active). Importantly, little or no increase in the levels of the (unphosphorylated) H2AX histone variant itself was detected in *MLL2* FC/FC cells (Fig. 3A), supporting the conclusion that the elevated γH2AX levels indeed signify DNA damage, as expected. Together, these data are consistent with the chromosome-level results presented in Figures 1 and 2: *MLL2* mutation results in DNA damage—marked by γH2AX, which can be mapped by ChIP-seq to high resolution—and appears to be particularly prevalent in a subset of genes. As a representative example, γH2AX density was clearly increased across the *HIST1H1D* gene in *MLL2* FC/FC cells (Fig. 3B, left, top two panels; see also Supplemental Fig. S7A).

Because MLL2 interacts with elongation factor RECQL5, we also characterized transcript elongation and transcription stress in particular. For this purpose, we combined RNAPII ChIP-seq with genome-wide nuclear run-on sequencing (GRO-seq) analysis. Indeed, RNAPII ChIP-seq data can be difficult to interpret in isolation. For example, accumulation of RNAPII in the transcribed region of a gene can be explained in two different ways: by increased rates of transcriptional initiation (and thus elevated transcription/RNAPII levels) or, in stark contrast, by “slow elongation” such as that caused by transcription stress (see Ehrensberger et al. 2013 for details). Crucially, GRO-seq data can distinguish between these possibilities. Indeed, if the rise in RNAPII ChIP density inside a gene is due to higher initiation rates, then nascent transcription (the GRO signal) should also be elevated. Otherwise, it must be due to slow elongation/transcription stress (referred to here as transcription stress for simplicity). To identify genes with transcription stress in MLL2 FC/FC cells, we thus selected genes that had elevated levels of RNAPII by ChIP-seq but did not have a correlating increase in nascent transcription—i.e., GRO-seq—levels. As a representative example, RNAPII occupancy was clearly increased across the *HIST1H1D* gene in *MLL2* FC/FC cells, while nascent transcription was reduced (Fig. 3B, four bottom panels; see also Supplemental Fig. S7A). We confirmed the sequencing data at this and a couple of other genes by independent ChIP-qPCR experiments (Supplemental Fig. S7B).

In an initial attempt to address whether genes experiencing DNA damage also showed transcription stress, we now focused on the promoter-proximal region (transcription start site [TSS] ± 2 kb) of the 414 genes that had the highest levels of DNA damage (γH2AX) in *MLL2* FC/FC cells. Interestingly, not only γH2AX but also RNAPII occupancy was elevated in this region in MLL2 FC/FC cells and particularly so in the transcribed region from 500 base pairs (bp) downstream from the TSS, in the region where processive transcript elongation gets under way (Fig. 3C,D, see stippled boxes). Remarkably, however, although the mean RNAPII occupancy was clearly increased in the FC/FC cells (Fig. 3D), the level of nascent RNAPII transcription remained the same or was slightly decreased (Fig. 3E). A similar trend, but to a much smaller extent, was observed in active genes in general but without a noticeable increase in γH2AX levels (Supplemental Fig. S8A–C). The uncoupling of RNAPII occupancy and nascent mRNA production strongly indicates that transcript elongation is perturbed in the absence of MLL2, correlating with DNA damage at the most affected genes. As slow elongation will not normally affect gene expression levels (Ehrensberger et al. 2013), this is also in agreement with the finding that *MLL2* mutation affects the dynamics of transcription but not steady-state levels of mRNA expression at these genes.

### Increased transcription stress is associated with DNA damage

**Figure 4.**
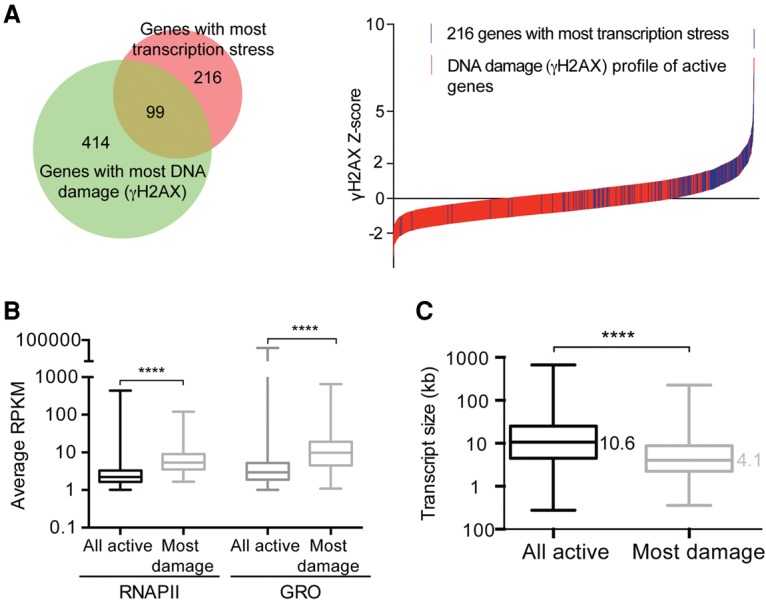
Genes with transcription stress and genes with DNA damage overlap. (*A*, *left*) Forty-six percent of the genes with the most transcription stress have high levels of DNA damage (γH2AX). (*Right*) The γH2AX *Z*-scores of all active genes were plotted (red), with the 216 genes with most transcription stress indicated in blue. (*B*) Genes with high levels of DNA damage in *MLL2* FC/FC cells are generally characterized by high levels of RNAPII and GRO compared with all actively transcribed genes. (*C*) Genes with high levels of DNA damage in *MLL2* FC/FC cells are generally short compared with all actively transcribed genes. The whiskers denote minimum to maximum. *P*-values were determined by *t*-test.

To more generally examine the connection between DNA damage and slow elongation/transcription stress in *MLL2* FC/FC cells, the extent of overlap between them was analyzed. As indicated above, we defined “transcription stress genes” as those with a RNAPII ChIP-seq ratio of >1 and a GRO-seq ratio of <1 in *MLL2* FC/FC cells compared with the F/F controls. There were 2158 such transcription stress genes. Remarkably, among the 216 (10%) genes with the highest level of transcription stress, 99 (46%) were also found among the 414 that had the most DNA damage (Fig. 4A, left). We also compared the ranked lists of transcription stress genes and genes with elevated DNA damage. As expected, genes with high transcription stress were significantly enriched at the top of the list of genes with DNA damage (*P*-value = 1.4 × 10^−60^, Kolmogorov-Smirnoff test) (see also Fig. 4A, right). Together, these data indicate that transcription stress in *MLL2* mutated cells is accompanied by DNA damage.

Bioinformatic analysis of the group of 414 genes with the most DNA damage in *MLL2* FC/FC cells showed that they are highly transcribed (Fig. 4B) and small in size, with a median length of only 4.1 kb versus 10.6 kb for all active genes in these cells (Fig. 4C). Not surprisingly, similar numbers characterized the 216 genes with the most transcription stress, which had a median length of 3.9 kb (Supplemental Fig. S8D). We note that, as the affected genes were generally short, the area of elevated DNA damage (γH2AX) analyzed above (Fig. 3) in effect represents a substantial proportion of the transcribed region of these genes. It is of particular note that 26 of the 414 genes with high levels of damage represent one of the 61 histone genes expressed in these cells; i.e., >40% of histone genes had highly elevated levels of DNA damage (γH2AX) when *MLL2* was mutated (*P* = 1.8 × 10^−11^, hypergeometric test). This interesting finding is explored in more detail below.

We also investigated whether the genes with the most DNA damage in *MLL2* mutated cells overlapped with known sites of genome instability. Remarkably, no less than 25% (105 of 414) of the genes with the most DNA damage and 33% (71 of 216) of the transcription stress genes in *MLL2* FC/FC cells overlapped with a class of cancer-associated chromosome fragile sites recently named ERFSs (Barlow et al. 2013). The probability of this being a chance event is 5.5 × 10^−5^ and 7.34 × 10^−8^, respectively (hypergeometric test). Replication programs are cell type-specific (Letessier et al. 2011), and, while MLL2-related DNA damage (γH2AX) and transcription stress were mapped in fibroblasts in the present study, the ERFSs were mapped in lymphocytes (Barlow et al. 2013), making the overlap all the more noteworthy.

We conclude that *MLL2* mutation results in transcription stress and DNA damage in short, highly active genes that overlap with previously mapped, cancer-associated ERFSs.

### MLL2 mutation affects RNAPII-associated histone methylation but not general histone methylation

**Figure 5.**
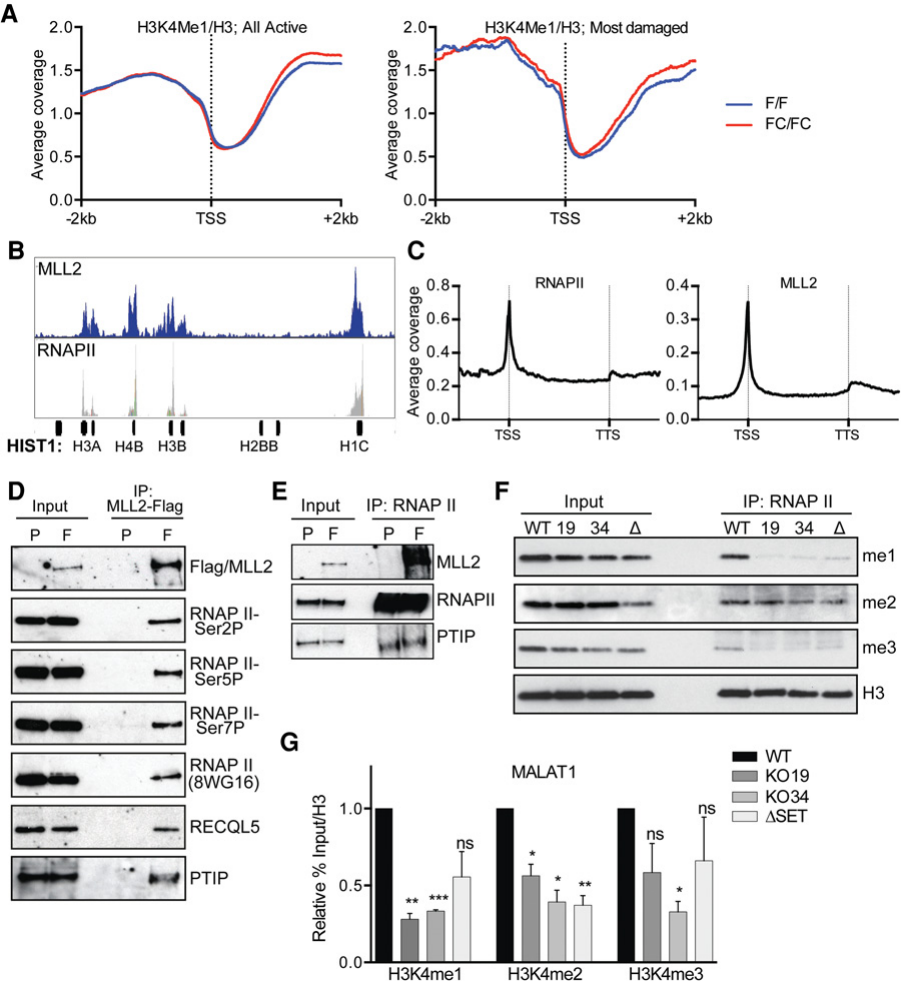
MLL2 associates with RNAPII. (*A*, *left*) Profiles of H3K4me1, normalized to H3, of all active genes in MEFs. (*Right*) The same, but for the genes with the most DNA damage in MLL2 FC/FC cells. (*B*) MLL2 and RNAPII are present at *HIST1* genes. (*C*) ChIP-seq meta-analysis of RNAPII and MLL2 gene profiles in HCT116 cells. (*B*,*C*) Original data from ENCODE (The ENCODE Project Consortium, 2012) and Hu et al. (2013). (*D*) MLL2-Flag coimmunoprecipitation (co-IP) experiments with HCT116 nuclear extracts. (*E*) As in *D*, but RNAPII immunoprecipitation (RNAPII-IP). The input represents 10% of the extract used for the immunoprecipitations, except for the RNAPII immunoprecipitations, where it was 1%. Parental cells (P) without MLL2-Flag were used as control. (*F*) RNAPII coimmunoprecipitated from HCT116 high-salt-extracted nuclear extracts and Western with antibodies against methylated H3K4 or H3 as indicated. (*G*) ChIP/reChIP experiments using HCT116 cells at the *MALAT1* locus. Primary ChIP was performed using anti-RNAPII antibodies; reChIP was with antibodies against methylated H3K4 or H3 as indicated. Error bars represent SEM. *n* = 2. *P*-values were determined by *t*-test. (WT) Parental cell line; (19) KO19; (34) KO34; (Δ) ΔSET.

Given that MLL2 is a histone methyltransferase, we now investigated whether the genes had measurable decreases in H3K4 methylation in *MLL2* FC/FC cells. Little if any difference in histone H3K4 methylation was observed across all genes between FC/FC and F/F cells (Fig. 5A, left; Supplemental Fig. S9A,B, left), and mono-, di-, and trimethylation were actually marginally elevated rather than decreased at the 414 most damaged genes in *MLL2* FC/FC cells (Fig. 5A, right; Supplemental Fig. S9A,B, right). In agreement with these data, others have previously reported that no decrease in histone methylation levels is observed in human and *Drosophila* cells lacking MLL2 (Herz et al. 2012; Hu et al. 2013).

Consistent with recent studies showing that *MLL2* mutation affects H3K4 methylation at enhancers (Hu et al. 2013; Lee et al. 2013; Cheng et al. 2014), we did detect potential mouse enhancers and other regulatory elements where H3K4 mono- and dimethylation in particular were affected (Supplemental Fig. S9C), but γH2AX-, RNAPII-, and GRO-levels were not significantly affected in these locations (Supplemental Fig. S9D).

Given that histone methylation was not markedly decreased in the *MLL2* mutated cells, we investigated whether MLL2 is normally present at genes, such as the highly γH2AX-enriched histone genes. The genome-wide occupancy of MLL2 has previously been investigated by ChIP-seq in HCT116 cells (Hu et al. 2013). Moreover, the RNAPII ChIP-seq profile for this cell type is available through the ENCODE project (The ENCODE Project Consortium 2012). Remarkably, these data show that MLL2 is indeed detected at human histone genes, with its density distribution nicely mirroring that of RNAPII (Fig. 5B). We also used the same data sets to perform metagene analysis of RNAPII and MLL2 density, specifically over gene bodies. As expected, RNAPII showed the characteristic profile with a prominent peak over the TSS and a smaller peak at the transcription termination site (TTS) (Fig. 5C, left). Remarkably, however, the metaprofile of MLL2 was similar (Fig. 5C, right), supporting the conclusion that MLL2 is more directly involved in transcription than has previously been recognized.

Given these data, we now investigated whether MLL2 interacts with RNAPII as well as RECQL5. As expected from the mass spectroscopy analysis of RECQL5, MLL2 could indeed coimmunoprecipitate RECQL5 (Fig. 5D). Furthermore, MLL2 interacted with not only PTIP but also different phosphorylated forms of RNAPII and also unphosphorylated RNAPII (as detected by the 8WG16 antibody) (Fig. 5D). The reverse was also true, as RNAPII coprecipitated Flag-tagged MLL2 and PTIP (Fig. 5E). The interaction with RNAPII, combined with its genomic profile over gene regions, thus suggests that MLL2 should be viewed as a general transcription-associated factor.

Histone modification is known to be highly dynamic around the elongating polymerase (Venkatesh and Workman 2015). One way of explaining the surprising finding that steady-state levels of histone methylation are not decreased by *MLL2* mutation would thus be if MLL2 only affects the dynamics of histone H3K4 methylation as an individual RNAPII molecule moves across a gene (i.e., cotranscriptional histone methylation). To investigate this possibility, we again used the human cell lines in which *MLL2* was deleted or lacked the catalytic SET domain (Guo et al. 2012, 2013), immunoprecipitated human RNAPII, and performed histone H3K4 Western blots on the resulting material. Similar amounts of histone H3 were associated with RNAPII in wild-type and mutant cell lines (Fig. 5F, bottom panel). However, while histone H3 was mono-, di-, and trimethylated at Lys4 in wild-type cells, especially mono- and trimethylation on these nucleosomes were highly depleted in *MLL2* mutated cells (Fig. 5F), suggesting that transcription-associated histone methylation is indeed perturbed in these cells. To further expand on this finding, we performed ChIP/reChIP experiments, using anti-RNAPII antibodies for the first round of immunoprecipitation and then anti-histone H3 methylation antibodies for the second round of immunoprecipitation from the RNAPII-associated material. Although technically challenging, in this approach, we also observed a significant depletion of RNAPII-associated histone H3 methylation using the highly expressed and highly affected MALAT1 gene as proxy for the most MLL2-affected genes (Fig. 5G).

Together, these results indicate that MLL2 plays an important role in cotranscriptional histone methylation, affecting only the histones in the immediate surroundings of elongating RNAPII.

### Increased mutation burden in the absence of MLL2

We now returned to the finding that histone genes in particular were affected by *MLL2* mutation. Interestingly, in this connection, recent data indicate that up to 80% of patients with follicular lymphoma carry *MLL2* mutation (Li et al. 2014; Okosun et al. 2014), a notable example of a strong cancer driver mutation. Significantly, the same patient cohort also presented a high level of mutations in histone genes. Intriguingly, as described above, histone genes presented elevated levels of DNA damage (γH2AX) in the mouse *MLL2* FC/FC cells. This raised the possibility that *MLL2* mutation results in histone gene mutation in both mouse cell lines and human cancer; i.e., the histone mutations detected in follicular lymphoma might be caused by *MLL2* mutation.

**Figure 6.**
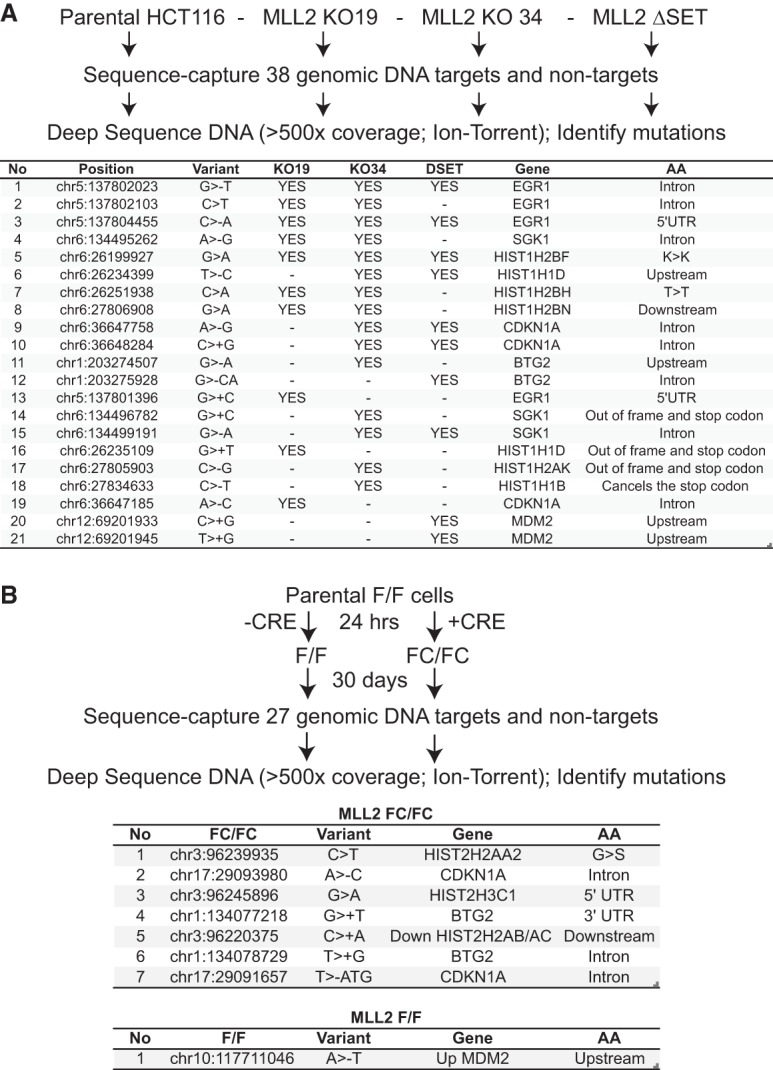
MLL2 disruption results in increased mutation rates in cell lines. (*A*, *top*) Experimental approach. (*Bottom*) Mutations detected by Ion Torrent deep sequencing in *MLL2* mutated HCT116 cells. (>) A substitution; (−) a deletion; (+) an insertion. The AA column indicates the relative position of the affected amino acid. (*B*, *top*) Experimental approach. (*Bottom*) Mutations detected in the FC/FC or parental F/F cells 30 d after *MLL2* excision.

To try to experimentally address this intriguing possibility, we first used the two human *MLL2* knockout cell lines and the ΔSET cell line, with the parental HCT116 cell line as control, to perform exome capture sequencing experiments to identify mutations that occurred upon *MLL2* deletion (Fig. 6A, top). Because of the sequencing depth required, these experiments were restricted to the histone genes that could be targeted with high coverage and a few other genes that were strongly affected in *MLL2* FC/FC cells. We also tested a short, highly transcribed control gene (RPLP0), which was largely unaffected by *MLL2* mutation. Remarkably, several mutations in target genes were independently established in the target genes in two out of three and sometimes even all three *MLL2* mutated human cell lines (Fig. 6A, bottom), while no mutations were detected in the RPLP0 control gene. It might be argued that these cell lines have simply randomly accumulated mutations. However, the fact that the same mutations were detected in the *MLL2* mutated cells (which were independently derived) (Guo et al. 2012, 2013) but not in the parental control cells strongly suggests that *MLL2* deletion led to the mutations. It is relevant to note that, as one might have expected, the majority of the established mutations gave rise to silent or intronic mutations or mutations upstream of/downstream from the ORF that would not be expected to affect the protein product. A limited number of mutations did result in truncated or improperly terminated proteins, though these were not among the recurrent mutations (Fig. 6A, bottom).

Finally, we also performed a similar experiment in mouse cells in which *MLL2* could be inducibly mutated. Here, we compared the mutations arising in cells that had the same genetic background prior to the experiment; i.e., that of the F/F cells. Cells were grown for 30 d with or without excision of *MLL2* on day 1 before analyzing the genetic profile by deep sequencing of the selected genes, as described above. Again, we considered the gene sequences found at the start of the experiment as baseline and then asked whether any mutations could be detected 30 d later, after ∼45 cell doublings (Fig. 6B). In agreement with an estimated mutation rate of ∼6 × 10^−8^ mutations per nucleotide site per generation for mouse cells in culture (Li et al. 1983), only one mutation was detected at the end of this growth period in the F/F control cells (*P* = 0.13, binomial test). In the *MLL2* FC/FC cells, however, we found seven mutations (*P* = 1.4 × 10^−10^), again indicating that *MLL2* mutation indeed results in a hypermutation phenotype.

Together, these data above strongly support the idea that loss of MLL2 function can induce mutations in genes, providing a likely explanation for previous observations in follicular lymphoma patients.

## Discussion

Recent genome-wide cancer studies have shown that MLL2 is a tumor suppressor that is mutated in a large number of different cancers. Indeed, in a recent survey of frequent cancer mutations, only a few well-studied drivers such as p53, B-raf, K-ras, and PTEN are more frequently mutated (https://www.intogen.org/search; Gonzalez-Perez et al. 2013). Here we show that MLL2 plays an important role in maintaining genomic stability, providing a new conceptual basis for understanding its widespread role in tumorigenesis. Using a variety of cell biological and genome-wide assays, we show that MLL2 inactivation results in genomic alterations (ranging from point mutations to gains and losses of genomic regions) and overall instability at the chromosomal level. Our data suggest that this can be explained at least partly by elevated RNAPII transcription stress in especially highly expressed, short genes, correlating with increased DNA damage in these genes.

### A role for MLL2 in ensuring genome stability

The data presented here strongly indicate that MLL2 is required to maintain genome stability. The evidence was obtained at a number of levels and in both mouse and human cells. For example, cells mutated for *MLL2* display a number of traits characteristic of genome instability, such as elevated levels of sister chromatid exchange, GCAs, 53BP1 foci, and micronuclei. In apparent agreement with our data, DNA damage and increased levels of reactive oxygen species have also recently been reported in bone marrow hematopoietic stem cells expressing the MLL-AF9 oncogene and lacking MLL2 (Santos et al. 2014). Moreover, CGH showed that a number of recurring chromosomal losses and gains are detected in *MLL2* mutated cells. Finally, as described in more detail below, *MLL2* mutation results in point mutations in genes.

Interestingly, similar but not identical observations on genome instability were made in mouse and human cells. The variations in instability observed between mouse fibroblasts and human colon carcinoma cells are unsurprising and, aside from the intrinsic dissimilarities between such cells, might conceivably be due to differences between stable human cell lines that have already adapted to life without MLL2 and the mouse cells, which are challenged by the sudden absence of it. Alternatively or additionally, the differences might be due to differential expression of other HMTs. Indeed, while only *MLL2* was targeted in our mouse cell model, the human *MLL2* knockout cells are known to also lack MLL3 expression (Guo et al. 2012). Under certain circumstances, MLL3 can substitute for MLL2 (Lee et al. 2009), and a role for other HMTs in compensation for MLL2 cannot be excluded.

In order to investigate exactly where in the genome DNA damage occurs, we performed γH2AX ChIP-seq experiments in normal and *MLL2* mutated cells. Interestingly, γH2AX can spread for megabases in response to a site-specific and persistent DNA double-strand break (Meier et al. 2007). In contrast, the spontaneous transcription-associated DNA damage described here does not give rise to such spreading. This might be because it does not give rise to persistent double-stranded breaks or because a lesion, even if it is a DNA double-strand break, will only arise at any single specific position in a tiny fraction of cells in a normal cell population. Indeed, our data show that genes with an elevated likelihood of instability display only modest γH2AX increases over the region. Importantly, in cells mutated for *MLL2*, we observed a significant increase in γH2AX and transcription stress in a subset of genes relative to normal cells. These genes were typically short and highly expressed. Indeed, it is possible that their preferential display of elevated levels of DNA damage (γH2AX) and point mutations is coupled to their transcription level; i.e., that *MLL2* mutation affects transcription stress at most genes, but its effects are more likely to be detected in highly active ones. In apparent agreement with this idea, the genes most affected by *MLL2* mutation overlap significantly with cancer-associated ERFSs, which are themselves enriched for short, highly active genes (Barlow et al. 2013).

Together, the available data thus strongly support the idea that, in the absence of MLL2, the process of transcription generally induces DNA damage and genome instability.

### A role for MLL2 in histone methylation during transcript elongation

Recent studies in human, mouse, and *Drosophila* cells show that MLL2 or its *Drosophila* homolog, Trithorax-related (TRR), is present at the TSS of actively transcribed genes (Herz et al. 2012; Hu et al. 2013; Lee et al. 2013). Strikingly, our metagene analysis of previous data (The ENCODE Project Consortium 2012; Hu et al. 2013) shows that RNAPII and MLL2 actually have very similar occupancy profiles across genes. In agreement with this, we found that *MLL2* mutation affects transcript elongation, in particular at highly active genes: In the absence of MLL2, RNAPII density increases in the transcribed region without concomitant increases in nascent RNA synthesis, indicating slow elongation and/or transcription stress. Moreover, MLL2 interacts with RNAPII and RECQL5, which plays an important role in transcriptional elongation genome-wide (Saponaro et al. 2014). MLL2 is thus part of the protein machinery that ensures smooth transcription and transcript elongation in particular.

Although it is well established that MLL2 is a histone methyltransferase, we were unable to detect marked changes in H3K4 methylation either globally or at the genes with most DNA damage in *MLL2* mutated cells. This is in line with previous work showing that decreases in histone H3K4 methylation, primarily mono- and dimethylation, can be detected only at enhancers in *MLL2* mutated human cells (Hu et al. 2013; Lee et al. 2013; Cheng et al. 2014). Remarkably, however, *MLL2* mutation greatly affects H3K4 methylation of RNAPII-associated nucleosomes, as shown both by RNAPII coprecipitation and ChIP analysis. Together with the realization that the MLL2 profile across genes resembles that of RNAPII, this indicates a role for MLL2 in cotranscriptional histone H3K4 methylation. Actually, it is tempting to speculate that the H3K4 methylation marks typifying enhancers are established by MLL2 and recruited there by RNAPII transcribing enhancer RNA and that the differences in steady-state histone marks between enhancers and gene regions are due to, for example, promoter-dependent loading of proteins that ensure dynamic histone modification.

In any case, it is an obvious possibility that lack of cotranscriptional histone methylation resulting from *MLL2* mutation causes problems for transcript elongation, especially at highly active genes; that this in turn results in transcription stress (RNAPII backtracking in particular); and that the ensuing clashes with replication forks then lead to genome instability, including mutations in genes. Alternatively, or additionally, the change in histone methylation dynamics in genes might in itself cause problems for the replication machinery, again giving rise to mutation and genome instability.

It is also worth noting that some aspects of genome instability observed upon *MLL2* mutation were transient. To the best of our knowledge, such instability followed by relative stabilization has not previously been observed. In this connection, we note that the small but consistent relative increase in histone methylation at the genes with the most DNA damage in our mouse cells and in human cells (Hu et al. 2013) suggests that other HMTs can (over)compensate for its absence. The mechanisms that compensate for the absence of MLL2 might take time to establish, which might in turn explain why certain aspects of genome instability, such as 53BP1 foci and micronuclei, were seen only in the immediate wake of loosing MLL2 but not after longer cell growth. The need for such compensation—in all likelihood by other HMTs associating with RNAPII—might also help explain why mere mutation of the SET domain of MLL2 appears to be significantly more genome-destabilizing than a complete loss of MLL2.

### MLL2 mutation and the implications for cancer

It was previously suggested that MLL2 might impact cancer through its effect on enhancers (Herz et al. 2014). Without contradicting previous studies, we propose an additional way in which MLL2 may broadly impact carcinogenesis. We thus favor a model in which MLL2 inactivation, although likely not in itself essential for tumorigenesis (Issaeva et al. 2007), drives tumor evolution through the gains and losses of genomic regions and/or increased gene mutation; in other words, through a wave of genomic instability, particularly in active genes, fueling cancer evolution and heterogeneity. We believe that this model can nicely explain why *MLL2* is mutated in so many different types of cancers.

*MLL2* is particularly highly mutated in follicular and diffuse large B-cell lymphoma (Pasqualucci et al. 2011; Lohr et al. 2012; Morin et al. 2013; Okosun et al. 2014). In these studies, mutations in histone genes were identified alongside those in *MLL2*. Remarkably, several histone genes were in the group of genes that showed the most DNA damage in our *MLL2* mutated cells: These had high levels of γH2AX and were typically characterized by slow RNAPII transcript elongation/transcription stress. This supports the idea that deletion of *MLL2* results in mutation in genes, including histone genes. We directly examined this possibility by deep sequencing several genes in normal and *MLL2* mutated cells. Gratifyingly, compared with controls, we detected several gene mutations in both human and mouse cell lines with *MLL2* mutation. Although these results were performed in cell lines, they are in strong support of the idea that *MLL2* mutation also caused the histone mutations observed in follicular and diffuse large B-cell lymphoma.

It is important to stress that the mutations that we detected are in all likelihood only the tip of the iceberg; i.e., they merely represent a few specific sites in highly expressed genes, which, in these particular cell lines, become mutated repeatedly enough to become detectable. In other cell types and contexts, other mutation sites in other genes might well be prevalent. Interestingly, whole-tumor genome sequencing data indicate that hot spots of mutation are enriched in the transcribed region within the first 2 kb after the TSS, starting to rise immediately upon the transit from transcriptional initiation to active elongation (Khodabakhshi et al. 2012). We suggest that MLL2 is particularly important to suppress such mutation, at least partly by reducing replication problems resulting from clashes with RNAPII undergoing transcription stress, and that, in its absence, cells therefore gain a “mutator” phenotype (Loeb 2001), which is particularly damaging because it preferentially targets active genes.

## Materials and methods

### Protein work

Immunoprecipitation and mass spectroscopy were performed as previously described (Aygun et al. 2008). Nuclear extracts were prepared using the Episeeker nuclear extraction kit (Abcam, ab113474) and treated with benzonase prior to immunoprecipitation and Western blotting. Antibodies to RECQL5 (Abcam, ab91422), Flag (Cell Signaling, 2368S), PTIP (Abcam, ab70434), and RNAPII (4H8 or 8WG16, Abcam) were used. The RNAPII-Ser2P and RNAPII-Ser7P antibodies were kind gifts from the Eick laboratory.

### Genome instability assays

Metaphase spreads, sister chromatid exchange assays, immunofluorescence staining, and counting of micronuclei were performed using standard assays and are described in the Supplemental Material. Mouse CGH experiments were performed using NimbleGen microarrays as previously described (Saponaro et al. 2014). Human CGH experiments were performed using Agilent microarrays according to the manufacturer's recommendations.

### Genome-wide analysis

ChIP, ChIP-seq, and GRO-seq experiments were performed as previously described (Saponaro et al. 2014). Ion Torrent sequencing and analysis were based on primers designed by the Ion Ampliseq designer (Life Technologies). The supporting data sets have been deposited in the Gene Expression Omnibus database (http://ncbi.nlm.nih.gov/geo) with accession number GSE73130.

For further details, please see the Supplemental Material.

## Supplementary Material

Supplemental Material
