# Supplementary material for: Mutation of cancer driver MLL2 results in transcription stress and genome instability
Source: Genes Dev. 2016 Feb 15;30(4):408–20. doi: 10.1101/gad.275453.115 (PMC4762426; doi:10.1101/gad.275453.115)
Supplement: Supplemental Material [file supp_30_4_408__index.html]

Supplemental Material 

# Mutation of cancer driver *MLL2* results in transcription stress and genome instability

## Supplemental Material

**Files in this Data Supplement:**

- Supp Figures S1-S9.pdf
- Supp Table S1.xlsx
- Supp Table S2.xlsx
- Supp Table S3.xlsx
- Supp Table S4.xlsx
- Supp Table S5.xlsx
