# Supplementary material for: Mutation of cancer driver MLL2 results in transcription stress and genome instability
Source: Genes Dev. 2016 Feb 15;30(4):408–20. doi: 10.1101/gad.275453.115 (PMC4762426; doi:10.1101/gad.275453.115)
Supplement: Supplemental Material [file supp_30.4.408_SuppFigsS1-S9.pdf]

A

RECQL5 IP:

Protein	Unique peptides
RECQL5	73
RBP1	61
RBP2	43
**MLL2**	**28**
INTS1	17
SPT5	15
SPT6	14
TERF2	13
SMC1A	11
INTS4	10

B

Protein	Unique peptides	
	EXP1	EXP2
RECQL5	24	20
**MLL2**	**16**	**14**
RBBP5	7	9
WDR5	4	2
ASH2L	5	4
PTIP	7	3

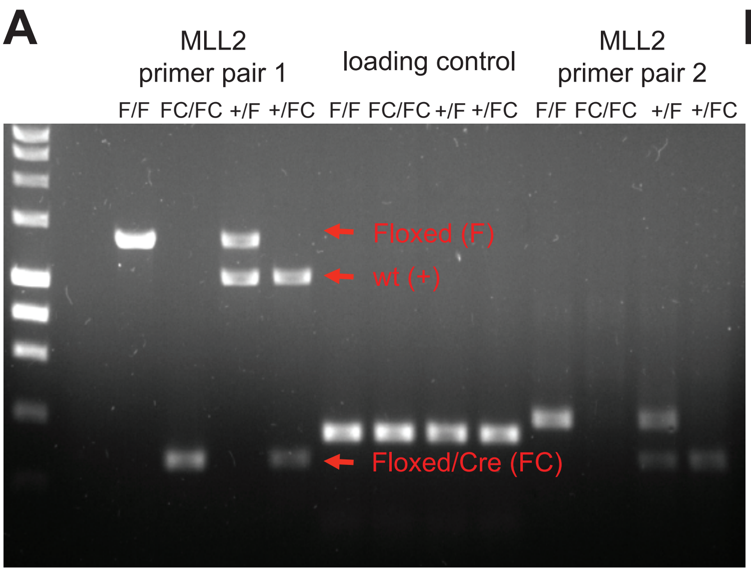

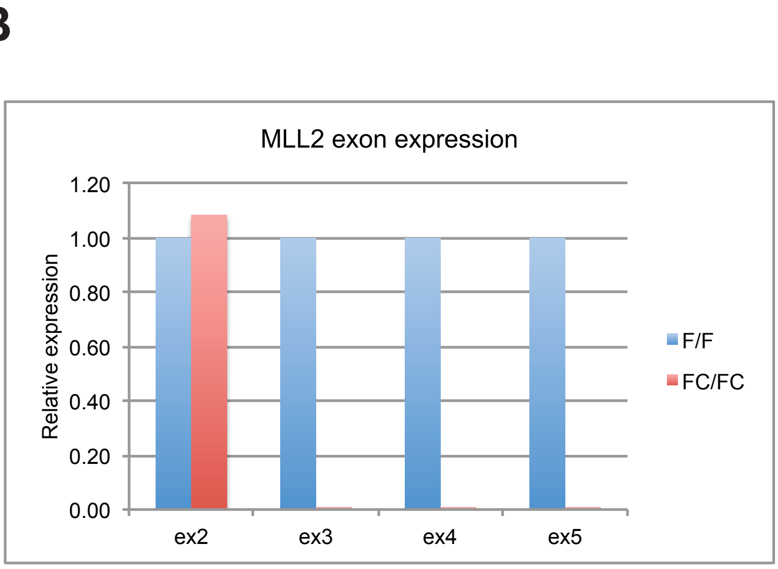

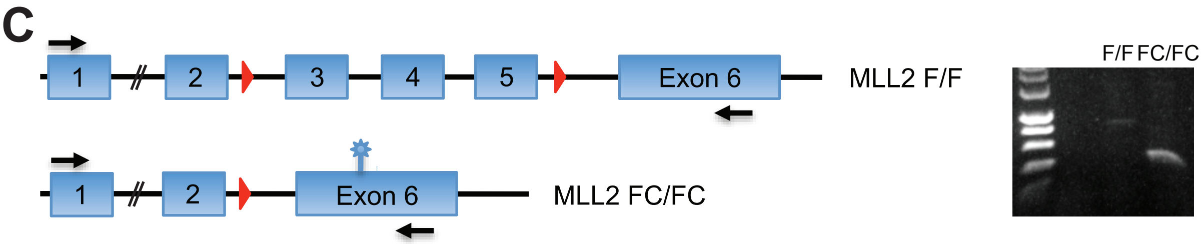

DNA sequence from PCR product of F/F

```
GCCAGTAGGGCGGCTGTGCTCGCTCTGGCGGTTGGAGGTCGGGGAGCGGCCCGGGCTCTGGCCATGTTCTC
GGATGAGGATTTCTGGATCGCCCTGTGAAGAGGTCTCCCCGAGAGGGCCCTGCCCAGTCGGAGAGAGGGAT
GGACAGCCAGAAGCCGCCTGCTGAAGATAAAGATTCAGACCCAGCAGCTGATGGACTTGCAGCCCCCGAGA
AGCCAGGTGCCACTGAGCCAGACCTTCCCATTCTGTGTATCGGGGAGGTCTCCGTCCCCGGTTCTGGGGGT
TCCAGGCCTCAGAAGCCTCCTCATGACTGCAGTAGGGGTCCAGCACGGCGTTGTGCTCTCTGTAACTGCGG
GGAGCCCGGTCTGCATGGGCAGAGGGAGCTGCAGCGCTTTGAGTTGCCATCTGACTGGCCCGGGTTTCCAG
TGGTACCCTCTGGGGGAAACTCAGGTCCCTGTGAGGCAGTGCTGCCCAAGGAGGACGCATCACAAATTGGT
TTCCCTGAGGGTCTTACGCCTGCCCACCTAGGAGAGCCTGGAGGGCACTGCTGGGCACATCATTGGTGTGC
AGCGTGGTCAGCAGGCGTATGGGGGCAGGAGGGCCCAGAACTATGTGGTGTGGACAAGGCCATCTTCTCAG
GGATCTCACAGCGCTGCTCCCACTGCGCCAGGTTCGGTGCCTCCGTTCCTTGCCGCTCACCCGGATGTTCC
CGGCTTTACCACTTTCGCTGTGCAACTGCCAGTGGTTCCTTCTTATCCATGAAAACGCTGCAGCTGCTCTG
CCCGTGGGGAGAGTGATAGAGGATGGCACATACATTCGAGAGGAGGGGACAACAATTTCCGTTATACCAAA
TGTCACGTCGAGACACTGGGACTTTACCGTGTACCTGCTGTTGTGTCCGTAACGCTCTCCAGAGTATATAA
ATCCGCATGGCAGAGCATTTGACATAGGCATCACGCCCCATGAGAGCGTCCGGAGAATGAAAGCCTCACTT
CAGCAGCCAGCGATAATCCTGACAGAAATCATTATTGCAGACGCTCTCTCTAGGATGCTCCCATCCTAGCT
GTCGTCATGTTTCCTAACTACAGCTACGATAATCTGATACCATGTCAGACAGATCTATCCCTGCTAGTATG
CCTGAACCATTGTCTAAGGTCCACGCAGTATAGATTCGATCTAGTAGGCCCGTTCTCATCGGACCGTGTAC
CTGATCCGTTCATACATCTGCGTCTGCTA
```

DNA sequence from PCR product of Fc/Fc

```
AGCCCAGTTGGGGCGCTGTGCTCGCTCTGGCGGTTGGAGGTCGGGGAGCGGCCCGGGCTCTGGCCATGTTC
TCGGATGAGGATTTCTGGATCGCCCTGTGAAGAGGTCTCCCCGAGAGGGCCCTGCCCAGTCGGAGAGAGGG
ATGGACAGCCAGAAGCCGCCTGCTGAAGATAAAGATTCAGACCCAGCAGCGCTGCTCCCACTGCGCCAGGT
TCGGTGCCTCCGTTCCTTGCCGCTCACCCGGATGTTCCCGGCTTTACCACTTTCCCTGTGCAACTGCCAGT
GGTTCCTTCTTATCCATGAAAACCCTGCAGCTGCTCTGCCCAAAACACAGTGATGGAACTGCACATAGTGG
GTTGGACGCATGCGTCTTCGCTTCGCACTGGGCACTCGGTCTTCGCATCGTTAGCTGGCAACGCATCTTTG
CCCCCAGAAGGTGCAGGGCTGCAAATGACCTCCAAATACCGCAGCGGCATGGGTGTGGTGTGGGACGCATA
TTCGGGCGCGCACACCGCAAACCTGGTTCCGGAGTTGATGGGCTTCTCCGCTGCTCAGCATGGACAGCTGA
GCAAAGAAATTGGCGAGGTTCGCGCACGAATCTATCGTAACCACCTCAACGGGACCGTTTTCCCGAACAAC
AATTTTCTGACCTGCTCGGGTGTCGTCAAGGTATGGCACCCGATCCACGCAAATACCACTGAGGTATGGAC
CTACGCCATGGTCGAAAAAGACATATCTTTCTATAAGATCTCCTACATATCTCAGTGCTGATGCAGAAGCA
GAAATGAAAAAACAAAAACCCCCATAGGGAGTGGGGGGTCCCCCCAGGCTGATGTAGGAAACTGCAGTCTT
CATTAACCCGAAAGGCTCGTGCGAAGACTGGCATTACGTGTTATGTGTTGTTTGCGGTGAGCGCTCCTCGA
GTTATAGACATTGCGCGGCGAGCGAATT
```

Exon1 Exon4
Exon2 Exon5
Exon3 Exon6
TGA -> stop

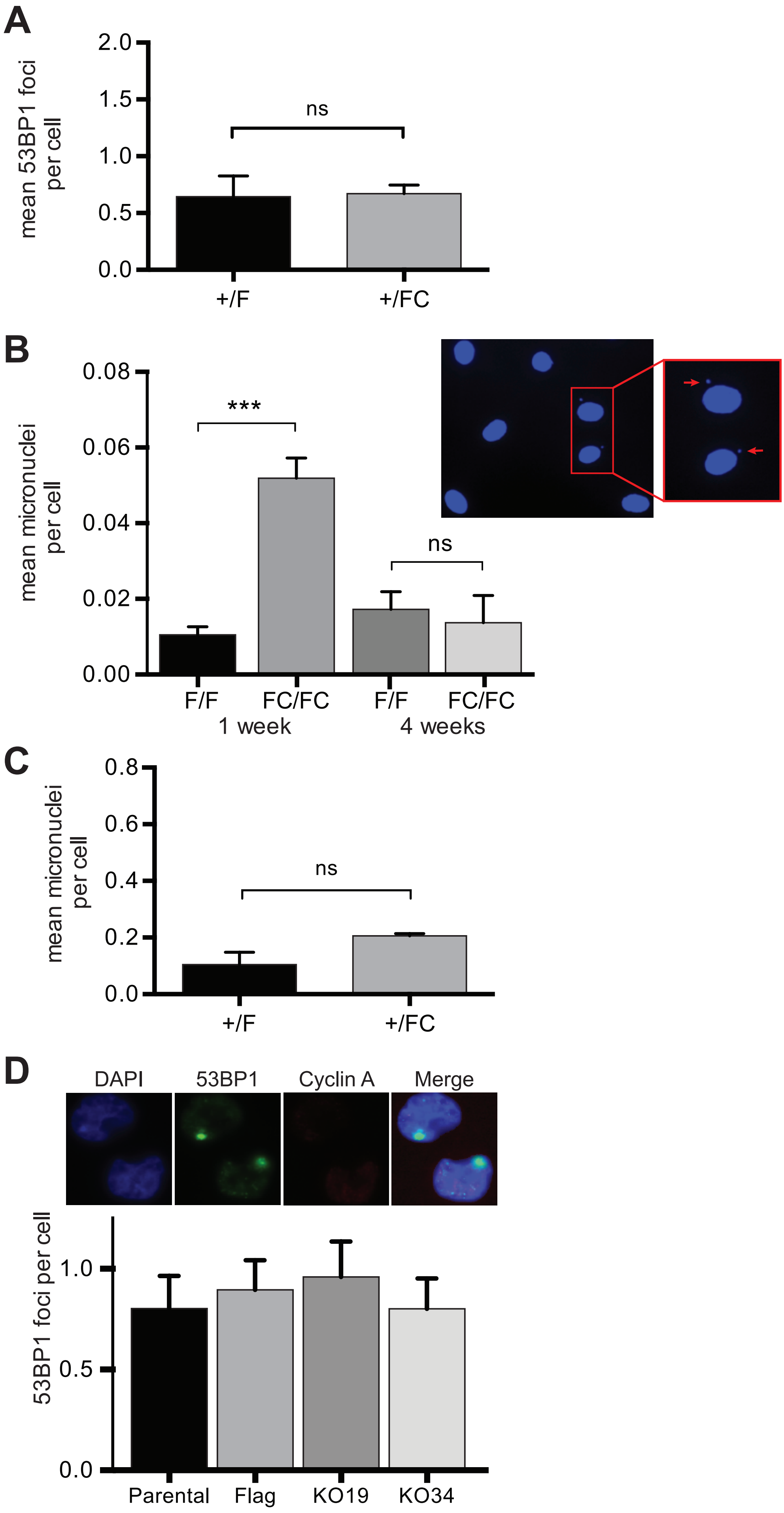
A
mean 53BP1 foci per cell
2.0
1.5
1.0
0.5
0.0
ns
+/F
+/FC
B
mean micronuclei per cell
0.08
0.06
0.04
0.02
0.00
***
ns
F/F
FC/FC
F/F
FC/FC
1 week
4 weeks
C
mean micronuclei per cell
0.8
0.6
0.4
0.2
0.0
ns
+/F
+/FC
D
DAPI
53BP1
Cyclin A
Merge
53BP1 foci per cell
1.0
0.5
0.0
Parental
Flag
KO19
KO34

A

CGH MEF cells MLL2 FC/FC

No	Chr	Start	Stop	Size (bp)	Cytoband	Aberration	Log2	Genes	Bacground
1	chr4	101,015,040	101,046,345	31,306	C6	Loss	-0.3453	YES	F/F
2	chr9	3,032,377	3,063,762	31,386	A1	Gain	0.4348	NO	F/F
3	chr11	23,514,589	23,582,875	68,287	A3.2	Loss	-0.2191	YES	F/F
4	chr18	15,056,594	15,067,015	10,422	A1	Loss	-0.2434	YES	F/F
5	chrX	166,578,982	166,648,585	69,604	F5	Gain	0.2046	NO	F/F
6	chrY	2,075,301	23,56,167	280,867	A1	Gain	0.2448	YES	F/F

B

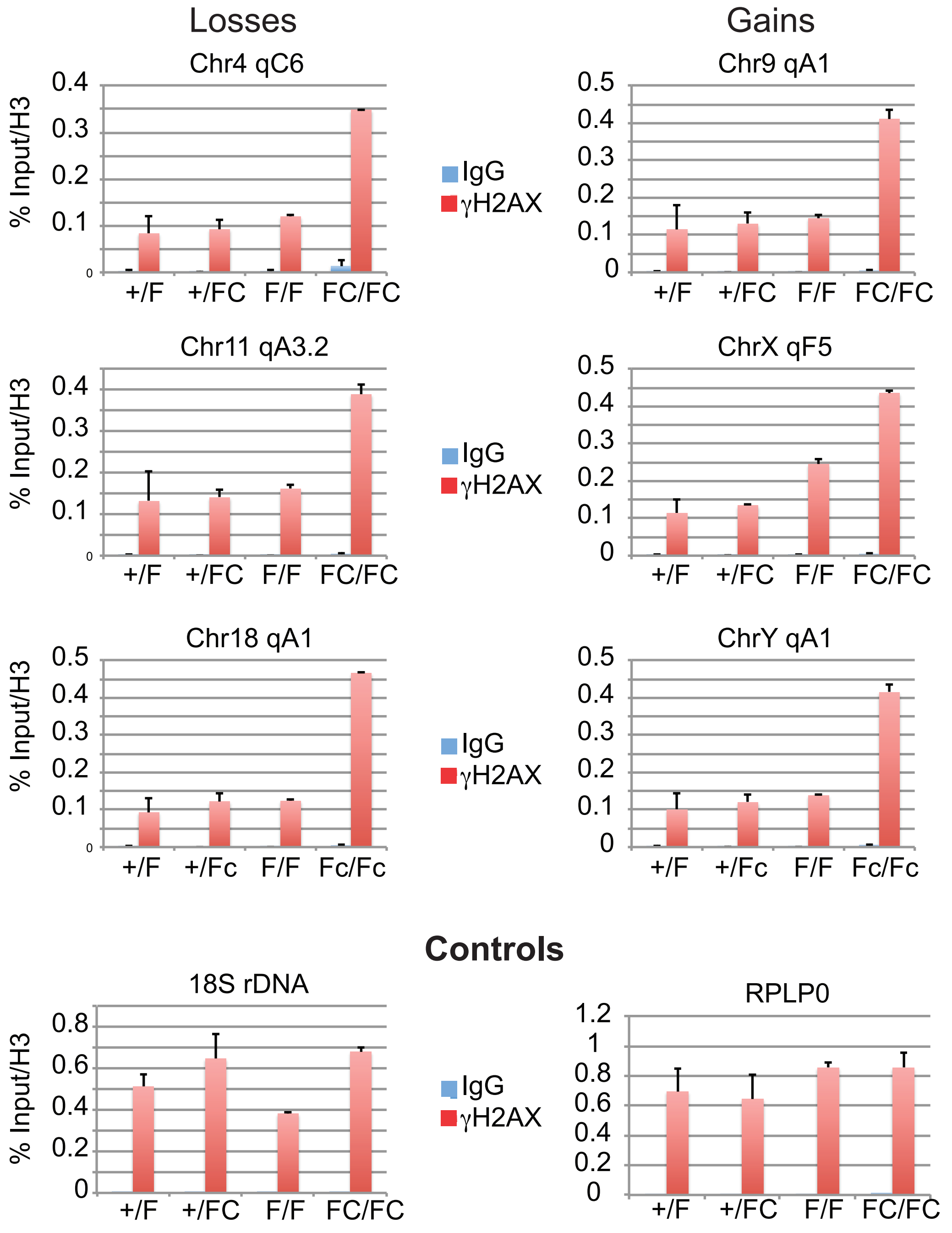

A

CGH - MLL2 KO19 and KO34 HCT116 cells

No	Chr	Start	Stop	Size bp	Cytoband	Aberration	Log2	Genes	Background	KO
1	chr1	28,166,614	28,306,969	140,355	p35.3	Gain	0.468	YES	Parental	19
2	chr2	200,168,994	200,280,889	111,895	q33.1	Loss	-0.778	YES	Parental	19
3	chr4	11,380,301	11,807,274	426,973	p15.33	Gain	0.924	YES	Flag	19/34
4	chr4	91,070,048	91,271,475	201,427	q22.1	Gain	0.505	YES	Parental	19/34
5	chr4	138,574,708	190,896,674	52,321,966	q28.3 - q35.2	Gain	0.522	YES	Parental/Flag	19/34
6	chr7	105,208,071	105,376,824	168,753	q22.3	Gain	0.432	YES	Parental/Flag	19/34
7	chr8	70,175,159	116,421,797	46,246,638	q13.2 - q23.3	Loss	-0.431	YES	Parental/Flag	19/34
8	chr12	34,250,295	34,417,451	167,156	p11.1	Loss	-0.470	NO	Parental	19/34
9	chr13	59,640,894	59,750,810	109,916	q21.2	Gain	4.217	NO	Flag	19/34
10	chr13	61,341,302	88,872,231	27,530,929	q21.2 - q31.2	Gain	0.966	YES	Flag	19/34
11	chr16	6,199,861	6,959,969	760,108	p13.3	Gain	1.115	YES	Parental/Flag	19/34
12	chr16	11,409,966	11,425,748	15,782	p13.13	Loss	-0.878	NO	Parental	19/34
13	chr20	13,982,937	14,062,307	79,370	p12.1	Gain	1.466	YES	Flag	19/34
14	chr21	14,513,884	48,095,856	33,581,972	q11.2 - q22.3	Gain	0.500	YES	Parental/Flag	19
15	chrX	96,438,867	96,707,566	268,699	q21.33	Gain	2.591	YES	Flag	19/34
16	chrX	107,527,827	108,295,113	767,286	q22.3	Loss	-1.823	YES	Parental/Flag	19/34
17	chrY	2,648,113	9,940,379	7,292,266	p11.31 - p11.2	Loss	0.934	YES	Parental/Flag	19/34
18	chrY	13,139,952	28,757,819	15,617,867	q11.1 - q11.23	Loss	0.906	YES	Parental/Flag	19/34

B

CGH - MLL2 ΔSET HCT116 cells

No	Chr	Start	Stop	Size bp	Cytoband	Aberration	Log2	Genes	Background
1	chr2	16,788,309	16,815,897	27,589	p24.2	Loss	-1.034	YES	Parental/Flag
2	chr2	99,860,478	99,919,064	58,587	q11.2	Loss	-0.449	YES	Flag
3	chr2	232,759,088	232,826,975	67,888	q37.1	Gain	0.606	YES	Parental/Flag
4	chr3	61,413,121	61,634,765	221,645	p14.2	Gain	0.530	YES	Parental/Flag
5	chr3	146,897,529	146,937,549	40,021	q24	Loss	-0.875	NO	Parental/Flag
6	chr4	11,391,438	11,807,274	415,837	p15.33	Gain	0.738	YES	Flag
7	chr4	13,427,395	13,750,482	323,088	p15.33	Gain	0.394	YES	Parental/Flag
8	chr4	169,643,796	169,785,063	141,268	q32.3	Gain	0.902	YES	Flag
9	chr4	190,498,388	190,678,708	180,321	q35.2	Gain	0.492	NO	Parental/Flag
10	chr5	26,142	8,872,480	8,846,339	p15.33 - p15.31	Gain	0.479	YES	Parental/Flag
11	chr5	153,944,047	160,883,034	6,938,988	q33.2 - q34	Loss	-0.734	YES	Parental/Flag
12	chr6	16,509,829	16,522,920	13,092	p22.3	Loss	-1.088	YES	Parental/Flag
13	chr6	137,127,900	137,147,511	19,612	q23.3	Gain	0.595	YES	Parental/Flag
14	chr7	62,452,971	73,713,374	11,260,404	q11.21 - q11.23	Loss	-0.694	YES	Parental/Flag
15	chr8	80,577,132	146,294,098	65,716,967	q21.13 - q24.3	Loss	-0.368	YES	Parental/Flag
16	chr9	204,193	24,797,812	10,124,563	p24.3 - p21.3	Loss	-0.792	YES	Parental/Flag
17	chr11	11,739,554	11,997,099	257,546	p15.3	Loss	-0.304	YES	Parental/Flag
18	chr13	29,035,924	29,067,636	31,713	q12.3	Gain	0.555	YES	Parental/Flag
19	chr13	59,640,894	59,750,810	109,917	q21.2	Loss	-2.412	NO	Parental
20	chr13	61,364,713	115,092,569	53,727,857	q21.2-q34	Loss	-0.637	YES	Parental/Flag
21	chr16	6,248,294	6,932,364	684,071	p13.3	Gain	0.395	YES	Flag
22	chr16	17,199,190	17,250,190	51,001	p12.3	Loss	-0.772	YES	Parental/Flag
23	chr18	54,562,509	54,967,423	404,915	q21.31	Loss	-0.459	YES	Flag
24	chr19	5,119,079	5,237,092	118,014	p13.3	Gain	0.802	YES	Flag
25	chr19	48,394,086	48,405,937	11,852	q13.33	Loss	-1.110	NO	Flag
26	chr19	58,719,082	58,770,722	51,641	q13.43	Loss	-0.739	YES	Parental/Flag
27	chr20	13,971,732	14,062,307	90,576	p12.1	Gain	0.851	YES	Flag
28	chrY	2,711,455	9,847,154	7,135,700	p11.31 - p11.2	Gain	0.405	YES	Flag
29	chrY	13,139,952	27,328,334	14,188,383	q11.1 - q11.23	Gain	0.435	YES	Flag

Kantidakis_FigS6

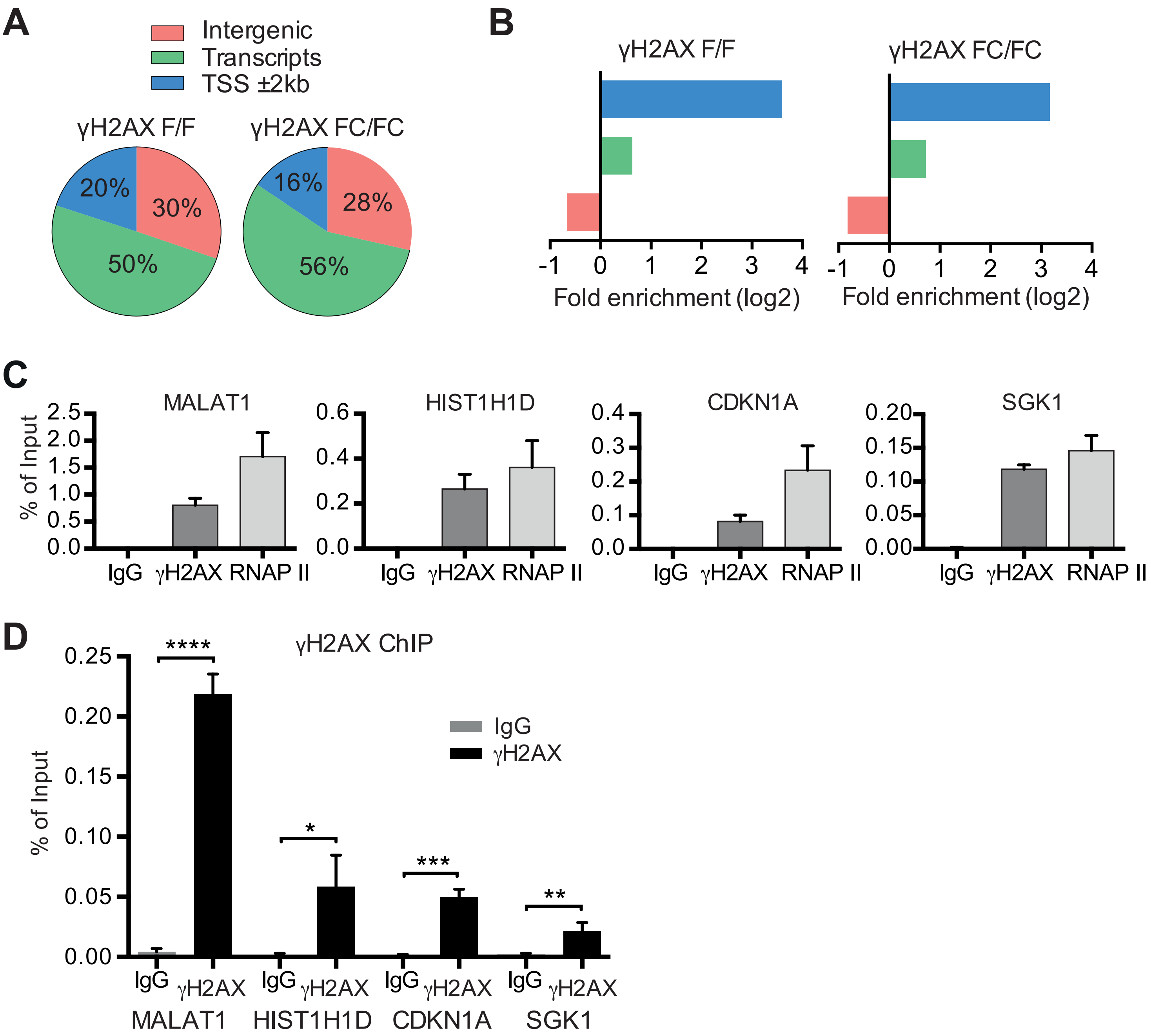

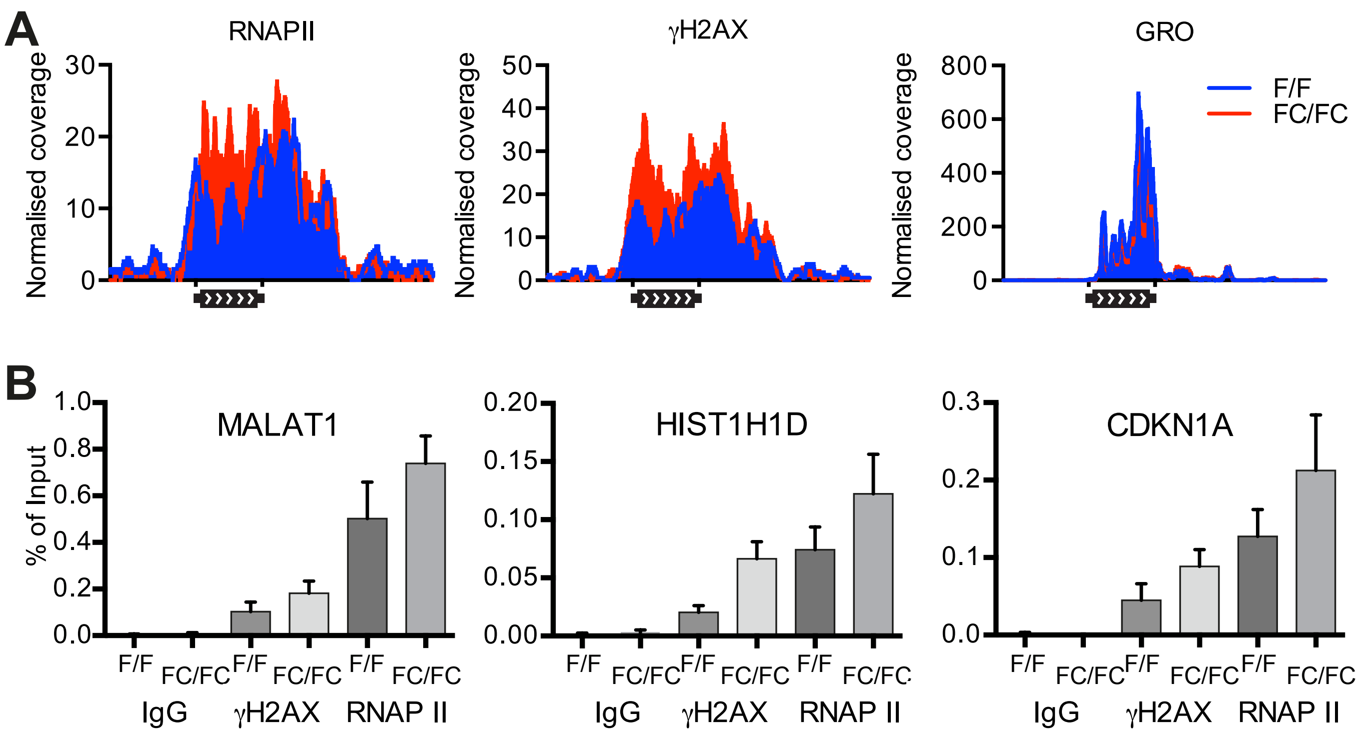
A
RNAPII
γH2AX
GRO
Normalised coverage
F/F
FC/FC
B
MALAT1
HIST1H1D
CDKN1A
% of Input
F/F
FC/FC
IgG
γH2AX
RNAP II

Kantidakis_FigS8

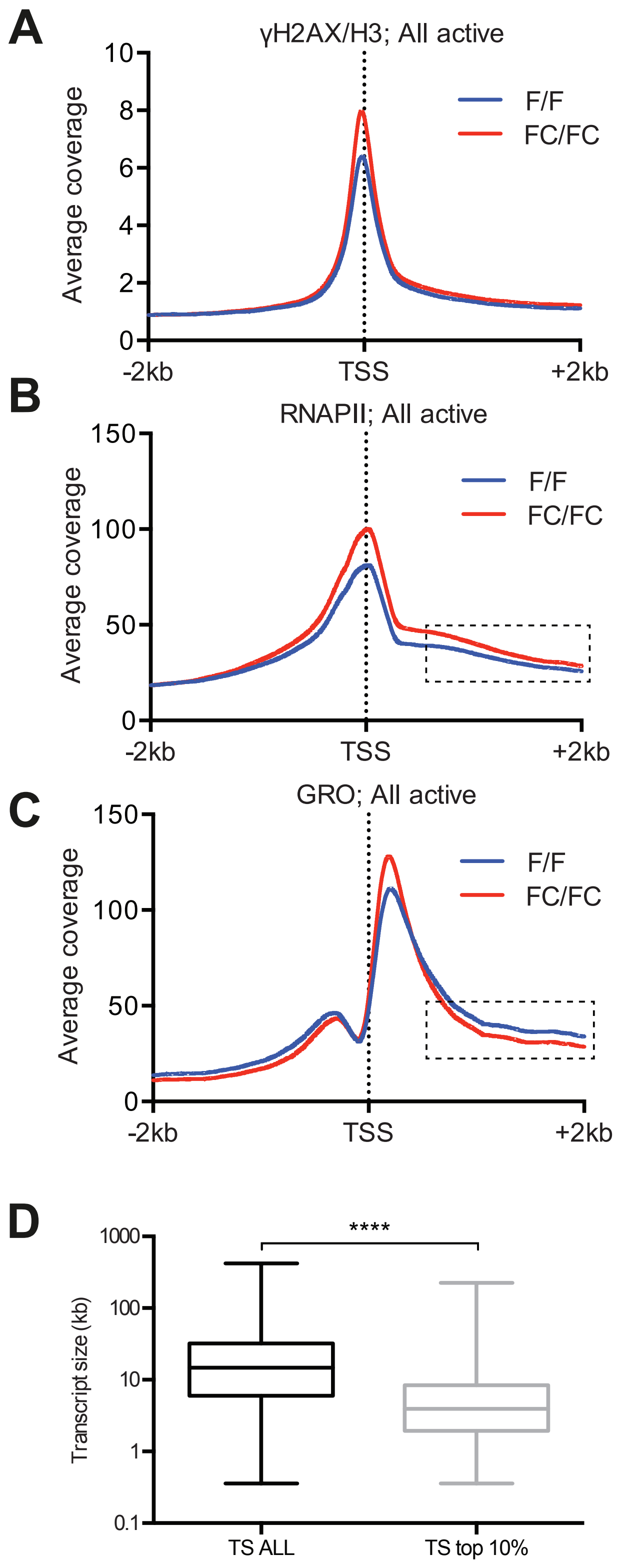

Kantidakis_FigS9

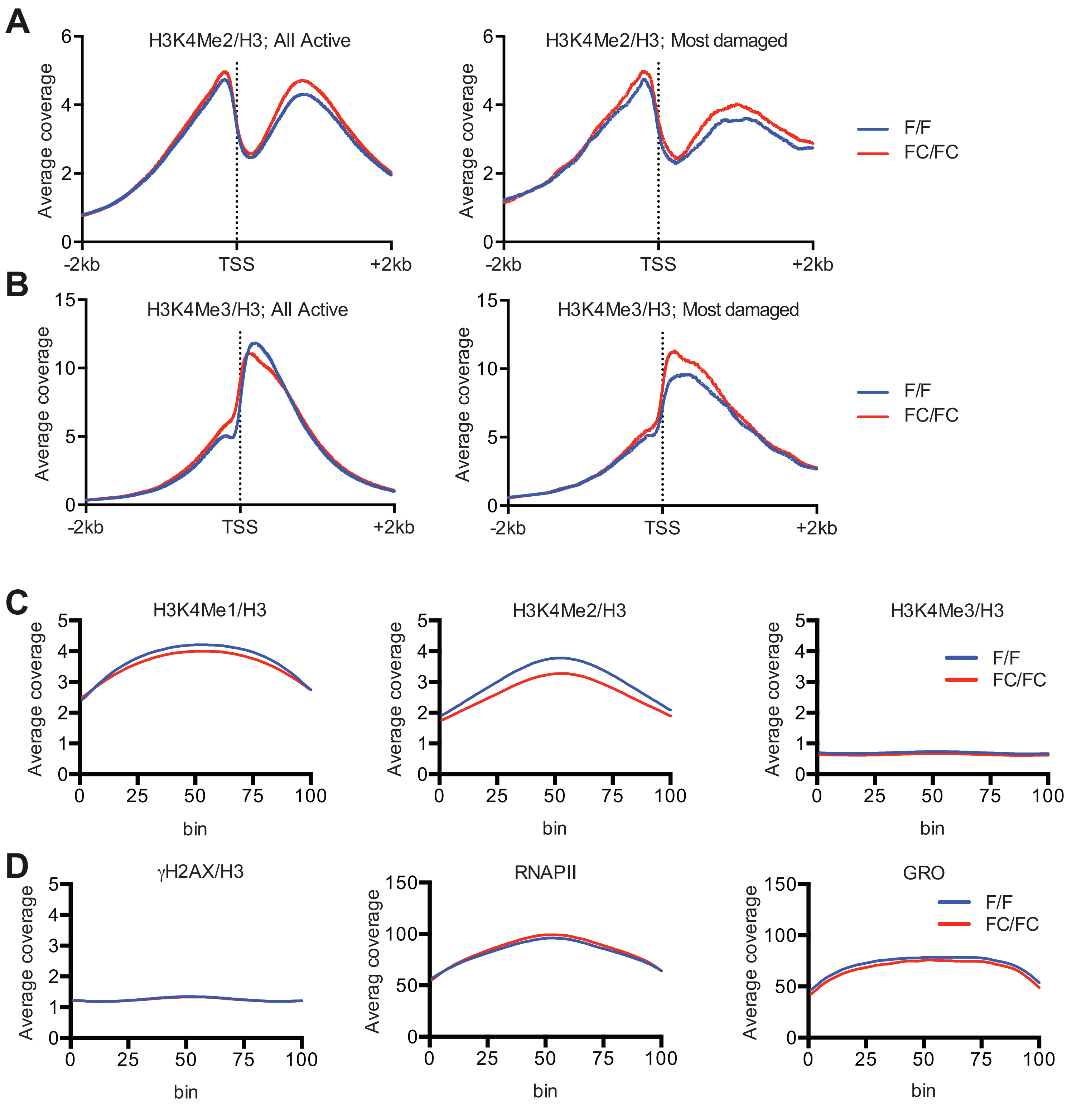

Supplementary Figure Legends

Figure S1. MLL2 associates with RNAPII and RECQL5. (**A**) and (**B**) Proteins detected by mass spectrometry after affinity-purification of chromatin-associated RECQL5. See Supplementary tables S1 and S2 for full list of proteins detected.

Figure S2. *MLL2* excision after tamoxifen treatment. (**A**) F/F or +/F cells were treated with 1 μM of hydroxyl-tamoxifen, or DMSO vehicle, for 24 hours. Genomic DNA PCR, with two different primer sets, confirms the excision of *MLL2*. (**B**) qPCR, 1 week after hydroxyl-tamoxifen treatment, indicates expression of *MLL2* exon 2, but diminished expression of the deleted exons, as expected. (**C**) Total RNA was isolated from F/F or FC/FC cells 1 week after hydroxyl-tamoxifen treatment and reverse-transcribed to cDNA. Primers based on exon 1 and exon 6, shown by black arrows, were used to PCR amplify exons 1-6. The red arrows indicate the location of the loxp sites. The diagram is not drawn to scale. The DNA was run in a gel (inset), purified and sequenced. The DNA sequencing shows the cDNA in F/F and FC/FC cells. Due to the deletion of *MLL2* exons 3-5, the recovered cDNA of FC/FC cells is shorter and results in a change of the reading frame, resulting in a stop codon (TGA). The different colors denote exons 1-6. The stop codon is shown in red background.

Figure S3. Immunofluorescence and micronuclei assays in mouse MEFs or human HCT116 cells. (**A**) 53BP1 foci quantification of mouse MLL2 +/F or +/FC cells one week after hydroxyl-tamoxifen treatment. The +/FC cells that still express

one *MLL2* allele, exhibit the same numbers of foci as the control +/F cells. n>600 cells for each cell line. (**B**) Micronuclei quantification of mouse *MLL2* F/F or FC/FC cells one or four weeks after hydroxyl-tamoxifen treatment. Red arrows in the image indicate micronuclei. n=3 independent experiments scoring >1000 cells in total for each cell line. (**C**) Micronuclei quantification of mouse *MLL2* +/F or +/FC cells one week after hydroxyl-tamoxifen treatment. The +/FC cells that still express one *MLL2* allele, do not exhibit a significant increase in the micronuclei numbers compared to the control +/F cells. n>600 cells for each cell line. (**D**) 53BP1 foci quantification of human HCT116 cells. The *MLL2* KO cells do not present increased number of foci compared to the controls in these stable cell lines. A typical image is shown (top panel). n>1000 cells for each cell line. p-values determined by Mann-Whitney test.

Figure S4. Comparative genomic hybridization in mouse MEF cells. (**A**) Genomic gains or losses found by comparative genomic hybridization (CGH) in FC/FC cells, compared to F/F cells, one or four weeks after *MLL2* excision. (**B**) γH2AX ChIP experiments at the proximity of the mapped gains/losses breakpoints from CGH, a week after MLL2 excision. The *18S rDNA* and *RPPL0* loci were used as extra controls.

Figure S5. Comparative genomic hybridization in human HCT116 cells. (**A**) Genomic gains or losses found by CGH in MLL2 KO19 or KO34 cells compared to the parental or MLL2-Flag control cells. (**B**) Genomic gains or losses found by CGH in MLL2 ΔSET cells compared to the parental or MLL2-Flag control cells.

Figure S6. ChIP analysis for γH2AX and RNAPII at specific genes. (**A**) γH2AX peak distribution over genomic features for F/F and FC/FC cells. (**B**) γH2AX enrichment analysis for F/F and FC/FC cells. (**C**) ChIP-qPCR experiments confirm the presence of γH2AX and RNAPII at specific genes. Anti-γH2AX antibody (ab2893, Abcam) and anti-RNAPII (4H8, Abcam) were used. (**D**) ChIP-qPCR experiments with an alternative anti-γH2AX antibody confirm the presence of γH2AX at specific genes. Anti-γH2AX (05-636; Milipore) was used. Error bars represent SEM. n=3. p-values determined by t-test.

Figure S7. γH2AX and RNAPII ChIP and GRO analysis in F/F or FC/FC cells. (**A**) Normalised coverage of RNAPII ChIP-Seq, γH2AX ChIP-Seq and GRO-Seq at the *HIST1H1D* gene for F/F and FC/FC cells. (**B**) ChIP-qPCR experiments confirm accumulation of γH2AX and RNAPII at FC/FC cells compared to F/F cells at specific genes. Error bars represent SEM. n=3.

Figure S8. γH2AX/H3, RNAPII ChIP-Seq and GRO-Seq profiles of all actively transcribed genes. Profiles for (**A**) γH2AX, normalized to H3, (**B**) RNAPII, and (**C**) GRO, for all actively transcribed genes. (**D**) The genes with most transcription stress are significantly shorter compared to all transcriptionally stressed genes. The whiskers denote min to max. p-values determined by t-test.

Figure S9. H3K4-methylation ChIP-Seq profiles at the TSS of actively transcribed genes or >5kb away. (**A**) Left, profiles of H3K4Me2, normalized to H3, of all active genes. Right, same as left, but for most damaged (γH2AX) genes (**B**) As in (A), but for H3K4Me3 (**C**) H3K4 methylation ChIP-Seq profiles at H3K4Me1 peaks more than 5kb from annotated TSS. (**D**) γH2AX/H3, RNAPII ChIP-Seq and GRO-Seq profiles at H3K4Me1 peaks more than 5kb from annotated TSS.

Supplemental Material and Methods

Cell lines

The F/F and +/F mouse embryonic fibroblasts (MEF) were immortalized using the 3T3 method. Cells were grown in DMEM (Gibco) supplemented with 10% FCS, 1% L-glutamine and 1% penicillin/streptomycin at 37°C with 5% CO_2. For *MLL2* excision, the cells were incubated with 1 μM of hydroxyl-tamoxifen (Sigma-Aldrich, H7904) for 24 hours. Cells were then washed twice with PBS before the addition of fresh medium. The cells were left to grow for 6 further days before harvesting, unless otherwise stated. The *MLL2* mouse BAC (RP23-458P18) was purchased from Children's Hospital Oakland Research Institute. The BAC was modified by inserting FLAP tags at the C-terminus and cloned into F/F cells as previously described (Lekomtsev et al. 2010). The clones used were selected to express one copy of the modified *MLL2* BAC.

Mass spectroscopy

Chromatin extracts were prepared from *RPB3*-Flag- or *RECQL5*-Flag- tagged human HEK293 cells. Flag immunoprecipitation and mass-spectroscopy were performed as previously described (Aygun et al. 2008).

Co-immunoprecipitations and Western Blotting

Nuclear extracts (NE) were prepared from human HCT116 cells by the Episeeker Nuclear extraction kit (Abcam, ab113474) according to manufacturer's instructions, but without using DTT. The samples were finally sonicated for 5 minutes at high intensity, using Bioruptor (Diagenode). For the high salt NE, the nuclear extraction buffer was complemented to a final 600mM

NaCl. At the last step the NE was treated with 50U benzonase per 10^6 cells for 30 minutes at room temperature. The NE extracts were quantified by the Bradford method and 500 µg of total protein were used per immunoprecipitation (IP). The high salt NE were diluted to a final 150mM salt, before proceeding. The NE was pre-cleared using 5 µg of rabbit or mouse IgGs with protein A or G Dynabeads (Life Technologies) for one hour, rotating at 4°C. 5 µg of the IP antibodies were then added to the pre-cleared NE and incubated, for two hours rotating at 4°C, with 40 µl (20 µl packed) of protein A or G Dynabeads (Life Technologies) or anti-Flag M2 magnetic beads (Sigma-Aldrich). Finally, the beads were washed six times with ice-cold wash buffer (40 mM Hepes [pH 7.8], 2 mM EDTA, 150 mM NaCl, and 0.1% CHAPS). To elute, SDS-loading buffer with DTT was added to the beads, which were then mixed and boiled for five minutes. The following antibodies were used for immunoprecipitations and Western Blotting (1:1000 dilution): anti-RECQL5 (Abcam, ab91422), anti-Flag (Cell Signaling, 2368S), anti-PTIP (Abcam, ab70434), anti-RNAPII (in house 4H8 or 8WG16), anti-H3 (Abcam, ab1791), anti-H3K4Me1 (Abcam, ab8895), anti-H3K4Me2 (Millipore, 07-030), anti-H3K4Me3 (Abcam, ab8580). The RNAPII-Ser2P and -Ser7P antibodies were a kind gift from the Eick lab (Munich, Germany).

Metaphases

Where stated, mouse MEF or human HCT116 cells were treated with 100 nM or 20 nM camptothecin (Sigma-Aldrich), respectively, or an equal volume of vehicle (DMSO) for 16 hours. The cells were then washed twice with PBS and left in culture with fresh medium for another 24 hours to reach confluency of ~70-80%.

0.2 µg/ml of colchicine were added to the cells for 3 hours, before they were harvested, washed twice with PBS and slowly resuspended in 5 ml pre-warmed 75 mM KCl. The cells were then incubated at 37°C for 10 minutes, collected by centrifugation, resuspended in 5 ml fixation buffer (Methanol 3:1 Acetic acid) and incubated at room temperature for 15 minutes. Two more rounds of resuspension and incubation in fixation buffer were performed, before the pellets were stored in fixation buffer at -20°C. Spreads were made from ~50 cm height to slides tilted by 45°. The slides were allowed to air-dry before stained in 7% Giemsa (v/v, in 10 mM PIPES ph 6.8) at RT for 30 minutes. The slides were then washed 3 times with water, air-dried and mounted. At least 100 metaphases per condition were scored.

Sister chromatid exchange assays

Sister chromatid exchange assays were performed as previously described (Bayani and Squire 2005) with cells cultured in 5 mg/ml BrdU (Sigma-Aldrich) for 40 hours followed by 0.2 µg/ml colchicine for 3 hours. 60 metaphases per condition were scored.

Immunofluorescence staining and micronuclei

Cells were grown on coverslips in six-well plates, fixed in 4% paraformaldehyde and processed as previously described (Kantidakis et al. 2010). The primary antibodies, used in 1:1000 dilution, were: anti-53BP1 (Abcam, ab36823) and anti-γH2AX (Abcam, ab2893 or Millipore, JBW301, 05-636). The secondary antibodies, anti-mouse or anti-rabbit Alexa Fluor 488/594, were purchased by Life Technologies. The coverslips were mounted using Vectashield with DAPI

(Vector Laboratories). For the micronuclei assays, the cells were processed as above and the nuclei were stained with DAPI. The 53BP1 foci were automatically quantified by Cell Profiler (Carpenter et al. 2006).

Comparative Genomic Hybridisation (CGH)

The QIAamp DNA kit (Qiagen) was used to isolate genomic DNA, according to the manufacturer's recommendation. The purity of the DNA was verified to be A260/280 >1.8, and A260/230 >1.9 by a NanoDrop spectrophotometer (Thermo Scientific). The integrity of DNA was also verified by agarose gel electrophoresis. The mouse CGH experiments were performed and analysed as previously described (Saponaro et al. 2014) using Nimblegen's (Roche) 3x720K mouse arrays. The human CGH experiments were performed using SurePrint G3 Human, 2x400K CGH Microarrays (Agilent) according to manufacturer's recommendation. The arrays were scanned by the Nimblegen MS200 micro-array scanning system (Nimblegen-Roche) and the data were analyzed using Agilent Cytogenomics 2.7.11.0 software following the default options.

Gene expression microarrays

Total RNA was extracted from MLL2 F/F and FC/FC cells 1 week after tamoxifen treatment using the RNeasy kit (Qiagen). Three independent biological replicates were employed. Total RNA was reverse transcribed using oligo-dT primers and the cDNA Synthesis System (Roche), according to the manufacturer's recommendations. cDNA labeling, hybridization, and washing of the microarrays were performed according to manufacturer's recommendations using the 12X135K human expression arrays (Nimblegen-Roche). The arrays

were scanned with the Nimblegen MS200 micro-array scanning system and images were processed with Nimblescan according to manufacturer's recommendations (Nimblegen-Roche). The data acquired were then processed using the DNAStar ArrayStar software (DNASTAR).

ChIP, ChIP/Re-ChIP, ChIP-Seq and GRO-Seq

ChIP, ChIP-Seq and GRO-Seq were performed and analyzed as previously described (Core et al. 2008; Saponaro et al. 2014). Three independent ChIP-Seq experiments for F/F as well as FC/FC cells were performed using antibodies against γH2AX, H2AX, H3K4Me1, H3K4Me2, H3K4Me3, H3, RNAPII and IgG. Two independent GRO-Seq experiments were also performed for F/F as well as FC/FC cells. In order to be able to perform a high-confidence analysis of the regions affected by *MLL2*-mutation, we restricted ourselves to look at those genes that - in both F/F and FC/FC cells - had high coverage for both RNAPII and γH2AX, as well as for GRO-Seq. A total of 56 ChIP-Seq and GRO-Seq experiments were employed. ChIP/Re-ChIP experiments were performed as previously described (Kantidakis and White 2010). Antibodies against RNAPII were employed in the first ChIP, while antibodies against H3, H3K4Me-1-2-3 were used in the secondary ChIP. The following antibodies were employed: anti-γH2AX (Abcam, ab2893), anti-H3 (Abcam, ab1791), anti-H2AX (Abcam, ab11175), anti-H3K4Me1 (Abcam, ab8895), anti-H3K4Me2 (Abcam, ab32356 or Millipore, 07-030), anti-H3K4Me3 (Abcam, ab8580), anti-mouse IgGs (Sigma-Aldrich, M8642), anti-rabbit IgGs (Sigma-Aldrich, R2004), anti-RNAPII (In house, 4H8), anti-BrdU (Santa Cruz Biotechnology, sc-IIB5). Anti-γH2AX (05-636; Milipore) was used for specificity controls in ChIP experiments.

ChIP-Seq and GRO-Seq analysis

Reads were aligned to the mm10 version of the mouse genome assembly using BWA v0.7.10 (Li and Durbin 2009) with default settings. Resultant SAM files were converted to BAM, sorted and indexed using Samtools v1.2 (Li et al. 2009). Mapped reads were extended 3' to a size of 200bp in a strand-specific manner. GRO-seq read strand information was reversed. A collection of genomic intervals representing Refseq transcripts mapped to the mm10 genomic assembly was downloaded from the UCSC refGene table.

RPKM and gene selection

Reads Per Kilobase per Million (RPKM) scores were calculated for each sample across all transcripts±500bp. These scores were used to generate a matrix of Spearman's correlation coefficients to visualize sample similarity. The Spearman's correlation coefficients ranged from 0.78 to 0.99 among the replicates, with a median of 0.93 among all. A set of actively transcribed genes showing a consistent RPKM >1 across each of the γH2AX, RNAPII and GRO-seq replicate samples for both F/F and FC/FC cells was selected. The threshold was chosen based on manual inspection of the aligned reads within IGV. Transcripts from duplicate genes and those shorter than 250 bp were discarded. The most damaged genes were chosen as the top 10% of (active) genes in terms of γH2AX FC/FC to F/F ratio, averaged across replicates.

Peak calling

Peaks were called against a control using MACS v1.4.2 (Zhang et al. 2008) with the following settings: --gsize (mm), --mfold (8,30), --pvalue (0.00001). Histone (H3K4Me1, H3K4Me2, H3K4Me3 and γH2AX) peaks were called against H3 controls, while RNAPII peaks were called against IgG controls. Peaks overlapping blacklisted regions identified by the ENCODE and modENCODE consortia were eliminated from further analysis (Consortium 2012).

Peak profiles >5kb from TSS

A collection of intervals representing the intersect of peaks called in all three H3K4Me1 replicates, and that lay more than 5kb from the nearest TSS, was used to profile F/F and FC/FC H3K4Me1 samples. Each peak was split into 100 equally sized bins. Sample read depth was normalized to 20 million and coverage within each bin of each peak was calculated. Subsequently, a mean across the same bin from all peaks were used to generate an average peak profile. This profile was averaged across biological replicates.

TSS profiles

Base pair level coverage was calculated across a set of unique Trancription Start Sites (TSS)+/-2kb, then normalized to a depth of 20 million reads. The mean normalized read-depth over each bp position was used to construct an average profile plot. Sample level profiles were subsequently averaged across biological replicates.

HCT116 cells

BigWig files representing MLL2 (GSM1240109) (Hu et al. 2013) and RNAPII (GSM803474) (Gertz et al. 2013) ChIP-seq data generated from HCT116 cells were downloaded from GEO. Coverages for 3804 human house-keeping genes, identified by (Eisenberg and Levanon 2013) as being expressed uniformly across a panel of tissues, were extracted and submitted to the same binning as described above. Each gene's binned coverage was converted to a z-score. A mean was taken across the two RNAPII replicates.

Enrichment

The genomic association tester (GAT) (Heger et al. 2013) was used to assess the enrichment of nucleotide overlaps between ChIP-Seq peaks and a range of genomic features: transcripts, TSS±2kb and intergenic regions. Merged BAM files representing aligned reads for each replicate group were used as input. The background workspace was defined as the genome minus any gap regions or regions unmappable with 51 bp reads. 10000 simulations were run for each sample. The division of peaks between the three feature sets was presented as a pie chart, while the log ratio of observed relative to the expected peaks per feature overlaps were presented as a barplot.

Ion Torrent sequencing and analysis

The Ion Ampliseq designer (Life Technologies) was used to design the primers. The human and mouse genes targeted are shown in table S5. The primers were purchased by Life Technologies and the libraries were prepared and sequenced according to the manufacturer’s instructions using the Ion PGM system (Life Technologies). Ion Torrent reads for mouse and human samples were mapped

to mm10 and hg19 reference sequences respectively, using the Ion Torrent optimized aligner Tmap (https://github.com/iontorrent/TMAP) with default settings. Putative somatic variants were called using Varscan 2.3.6 (Koboldt et al. 2012) with default settings, and post-processing buy the processSomatic jar included with the Varscan suite. Variants determined to be somatic were extracted. Candidate variants of interest were 'manually reviewed' by examination of the associated bam files using IGV (Robinson et al. 2011). For all somatic variants, total coverage and variant allele frequencies were extracted from the bam files for all samples and a Fisher's exact test applied to these data to supplement the SomaticP values provided by Varscan for those samples in which the variant is called. The variants were selected to have $p<0.001$ compared to the somaticP. All analyses were performed using R 3.0.2 except where noted.

References

Aygun O, Svejstrup J, Liu Y. 2008. A RECQ5-RNA polymerase II association identified by targeted proteomic analysis of human chromatin. *Proceedings of the National Academy of Sciences of the United States of America* **105**: 8580-8584.

Bayani J, Squire JA. 2005. Sister chromatid exchange. *Current protocols in cell biology / editorial board, Juan S Bonifacino [et al]* **Chapter 22**: Unit 22 27.

Carpenter AE, Jones TR, Lamprecht MR, Clarke C, Kang IH, Friman O, Guertin DA, Chang JH, Lindquist RA, Moffat J et al. 2006. CellProfiler: image analysis software for identifying and quantifying cell phenotypes. *Genome biology* **7**: R100.

Consortium EP. 2012. An integrated encyclopedia of DNA elements in the human genome. *Nature* **489**: 57-74.

Core LJ, Waterfall JJ, Lis JT. 2008. Nascent RNA Sequencing Reveals Widespread Pausing and Divergent Initiation at Human Promoters. *Science* **322**: 1845-1848.

Eisenberg E, Levanon EY. 2013. Human housekeeping genes, revisited. *Trends in genetics : TIG* **29**: 569-574.

Gertz J, Savic D, Varley KE, Partridge EC, Safi A, Jain P, Cooper GM, Reddy TE, Crawford GE, Myers RM. 2013. Distinct properties of cell-type-specific and shared transcription factor binding sites. *Molecular cell* **52**: 25-36.

Heger A, Webber C, Goodson M, Ponting CP, Lunter G. 2013. GAT: a simulation framework for testing the association of genomic intervals. *Bioinformatics* **29**: 2046-2048.

Hu D, Gao X, Morgan MA, Herz HM, Smith ER, Shilatifard A. 2013. The MLL3/MLL4 branches of the COMPASS family function as major histone H3K4 monomethylases at enhancers. *Molecular and cellular biology* **33**: 4745-4754.

Kantidakis T, Ramsbottom BA, Birch JL, Dowding SN, White RJ. 2010. mTOR associates with TFIIIC, is found at tRNA and 5S rRNA genes, and targets their repressor Maf1. *Proceedings of the National Academy of Sciences of the United States of America* **107**: 11823-11828.

Kantidakis T, White RJ. 2010. Dr1 (NC2) is present at tRNA genes and represses their transcription in human cells. *Nucleic acids research* **38**: 1228-1239.

Koboldt DC, Zhang Q, Larson DE, Shen D, McLellan MD, Lin L, Miller CA, Mardis ER, Ding L, Wilson RK. 2012. VarScan 2: somatic mutation and

copy number alteration discovery in cancer by exome sequencing. *Genome research* **22**: 568-576.

Lekomtsev S, Guizetti J, Pozniakovsky A, Gerlich DW, Petronczki M. 2010. Evidence that the tumor-suppressor protein BRCA2 does not regulate cytokinesis in human cells. *Journal of cell science* **123**: 1395-1400.

Li H, Durbin R. 2009. Fast and accurate short read alignment with Burrows-Wheeler transform. *Bioinformatics* **25**: 1754-1760.

Li H, Handsaker B, Wysoker A, Fennell T, Ruan J, Homer N, Marth G, Abecasis G, Durbin R, Proc GPD. 2009. The Sequence Alignment/Map format and SAMtools. *Bioinformatics* **25**: 2078-2079.

Robinson JT, Thorvaldsdottir H, Winckler W, Guttman M, Lander ES, Getz G, Mesirov JP. 2011. Integrative genomics viewer. *Nat Biotechnol* **29**: 24-26.

Saponaro M, Kantidakis T, Mitter R, Kelly GP, Heron M, Williams H, Soding J, Stewart A, Svejstrup JQ. 2014. RECQL5 controls transcript elongation and suppresses genome instability associated with transcription stress. *Cell* **157**: 1037-1049.

Zhang Y, Liu T, Meyer CA, Eeckhoute J, Johnson DS, Bernstein BE, Nusbaum C, Myers RM, Brown M, Li W et al. 2008. Model-based analysis of ChIP-Seq (MACS). *Genome biology* **9**: R137.
